# Multifunctional Asymmetric Bilayer Aerogels for Highly Efficient Electromagnetic Interference Shielding with Ultrahigh Electromagnetic Wave Absorption

**DOI:** 10.1007/s40820-025-01800-6

**Published:** 2025-06-12

**Authors:** Cheng-Zhang Qi, Peng Min, Xinfeng Zhou, Meng Jin, Xia Sun, Jianjun Wu, Yanjun Liu, Hao-Bin Zhang, Zhong-Zhen Yu

**Affiliations:** 1 State Key Laboratory of Organic-Inorganic Composites, Beijing University of Chemical Technology, Beijing, 100029 People’s Republic of China; 2 Center for Nanomaterials and Nanocomposites, College of Materials Science and Engineering, Beijing University of Chemical Technology, Beijing, 100029 People’s Republic of China; 3 Ningxiamogong Technology Co., Ltd, Yongning, 750100 People’s Republic of China

**Keywords:** Multifunctional bilayer aerogels, Electromagnetic interference shielding, MXene sheets, Graphene oxide, Infrared stealth and camouflage

## Abstract

Although multifunctional electromagnetic interference (EMI) shielding materials with ultrahigh electromagnetic wave absorption are highly required to solve increasingly serious electromagnetic radiation and pollution and meet multi-scenario applications, EMI shielding materials usually cause a lot of reflection and have a single function. To realize the broadband absorption-dominated EMI shielding via absorption–reflection–reabsorption mechanisms and the interference cancelation effect, multifunctional asymmetric bilayer aerogels are designed by sequential printing of a MXene-graphene oxide (MG) layer with a MG emulsion ink and a conductive MXene layer with a MXene ink and subsequent freeze-drying for generating and solidifying numerous pores in the aerogels. The top MG layer of the asymmetric bilayer aerogel optimizes impedance matching and achieves re-absorption, while the bottom MXene layer enhances the reflection of the incident electromagnetic waves. As a result, the asymmetric bilayer aerogel achieves an average absorption coefficient of 0.95 in the X-band and shows the tunable absorption ability to electromagnetic wave in the ultrawide band from 8.2 to 40 GHz. Finite element simulations substantiate the effectiveness of the asymmetric bilayer aerogel for electromagnetic wave absorption. The multifunctional bilayer aerogels exhibit hydrophobicity, thermal insulation and Joule heating capacities and are efficient in solar-thermal/electric heating, infrared stealth, and clean-up of spilled oil.

**Supplementary Information:**

The online version contains supplementary material available at 10.1007/s40820-025-01800-6.

## Highlights

- The multifunctional asymmetric bilayer MXene-graphene oxide (MG)-MXene aerogel with ultrahigh absorption electromagnetic interference (EMI) shielding is constructed by 3D printing and subsequent freeze-drying.
- The asymmetric bilayer aerogel achieves ultrahigh electromagnetic wave absorption coefficient of 0.95, and maintains a high EMI shielding effectiveness of over 100 dB.
- The multifunctional asymmetric aerogel with ultrahigh electromagnetic wave absorption, infrared camouflage, solar-thermal heating, and clean-up of organic solvents and spilled crude oil is developed for multi-scenario applications.

**Supplementary Information:**

The online version contains supplementary material available at 10.1007/s40820-025-01800-6.

## Graphical abstract

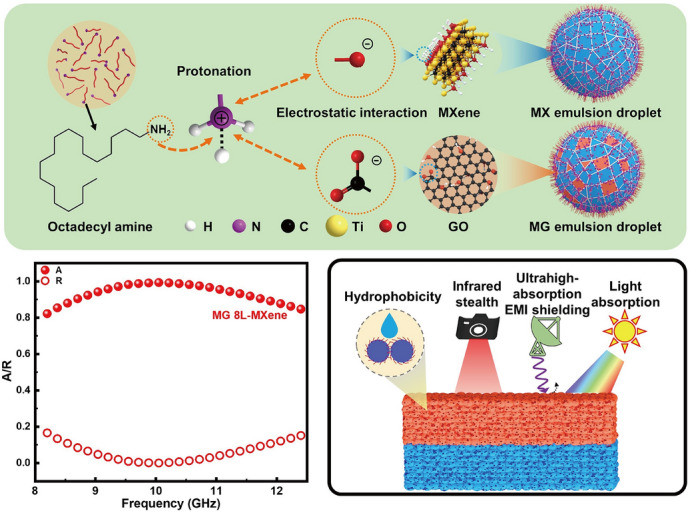

## Introduction

Serious electromagnetic interference (EMI) and radiation adversely affect normal operation and even cause severe malfunctions of electronic devices, and the electronic devices can also be easily detected by using advanced multi-spectrum detection techniques [1, 2]. Electrically conductive transition metal carbonitride (MXene) sheets with abundant surface functional groups have attracted much attention because of their remarkable electrical conductivity, high specific surface area, and ease of process in aqueous media [3, 4]. In particular, conductive MXene-based aerogels with high porosity, rich interfaces, and low density are promising for EMI shielding [5–8], electromagnetic wave absorption [9–11], thermal insulation [12], oil adsorption [13], energy storage [14, 15], and sensing [16, 17]. Currently, unifunctional MXene aerogels have their limitations in complex and variable application environments [18–20]. It is now imperative to design multifunctional MXene aerogels and expand their application scenarios [21, 22].

The structural design is crucial for achieving satisfactory EMI shielding performances of conductive MXene-based materials and architectures used in diverse scenarios [23, 24]. Generally, when incident electromagnetic waves encounter a conductive material surface via a propagating medium, the large impedance mismatch usually causes the reflection of a large proportion of the incident waves, thus causing secondary environmental pollution [25]. In particular, porous MXene aerogels can provide multiple reflection/scattering interfaces in their interiors and extend the propagation paths of electromagnetic waves, thus benefiting the enhancement in electromagnetic wave absorption. Gao et al. reported a silver microtubule/polydimethylsiloxane (PDMS) composite [26], in which the hollow structure of the silver microtubules could enhance multiple reflections of electromagnetic waves, interface polarization, and conduction loss, exhibiting absorption-dominated EMI shielding with an absorption coefficient (A) of 0.79 at 8.2 GHz.

The direct reflection of electromagnetic waves can be weakened by reducing the electrical conductivity of MXene-based materials to achieve impedance matching. Xue et al. fabricated a MXene/carbon nanotube/polyimide aerogel with a gradient conductive structure [27]. Its slightly conductive top layer reduced the electromagnetic wave reflection, while its highly conductive bottom layer serves as a reflective layer, resulting in a reduced reflection coefficient (R) of 0.23 in the X-band. Additionally, the addition of magnetic particles could help tune impedance matching, and the magnetic loss is beneficial for enhancing the electromagnetic wave absorption. Xu et al. prepared a multilayer composite foam and utilized reduced graphene oxide (RGO)@Fe_3_O_4_/waterborne polyurethane foam as an impedance-matching layer to facilitate the penetration of electromagnetic waves [28]. The directionally aligned porous structure of the multi-layer composite foam could mitigate the impedance mismatch, leading to an absorption coefficient of over 0.9 in the X-band. However, large-scale customization of multifunctional, ultralight, and broadband absorption-dominated EMI shielding materials remains challenging.

The newly emerged direct ink writing (DIW) technology enables inks to be stacked bottom-up in a programmed trajectory to customize 3D structures and provides high design freedom, efficient performance adjustment, and multifunction integration for customization design of MXene-based architectures before their mass-production. A satisfactory DIW ink should possess a suitable viscoelastic transition point and a shear-thinning feature, allowing the inks to be uniformly extruded from DIW needles. The rheological behavior of the inks plays a crucial role in 3D printing because high storage modulus and high ratio of storage modulus to loss modulus are usually required to ensure self-supporting and shape-preserving of the DIW inks [29, 30]. Therefore, DIW technology provides a convenient method for preparing asymmetric hierarchical structures in three-dimensional space.

Herein, we construct a multifunctional asymmetric bilayer aerogel with ultrahigh electromagnetic wave absorption and ultralow reflection by 3D printing to solve the problem of high reflection caused by impedance mismatch of electromagnetic shielding materials. The top MXene-graphene oxide (MG) layer of the bilayer aerogel optimizes impedance matching and achieves thickness matching of a quarter wavelength, while the bottom conductive MXene layer serves as an EMI shielding layer to impede the electromagnetic wave transmission and enhance the internal absorption. The emulsion inks for the 3D printing are generated on the basis of the electrostatic interaction of the organic solution of octadecyl amine (ODA) with the aqueous suspension of electronegative MXene and/or graphene oxide (GO) sheets at the oil/water (O/W) interfaces. During the preparation of the emulsion inks, the ODA with the hydrophilic amine groups can rapidly migrate from the oil phase to the O/W interfaces and be protonated, generating 2D surfactants with the electronegative sheets under electrostatic interactions and preventing the agglomeration of the droplets. Subsequently, an asymmetric MG-MXene bilayer aerogel is constructed by 3D printing or molding for achieving broadband absorption-dominated EMI shielding efficiency. The spherical porous microstructure in the aerogel can extend the propagation pathway of the electromagnetic waves, providing numerous reflecting and scattering interfaces. This strategic broadband absorption-dominated EMI shielding design can significantly reduce the reflection and transmission of the electromagnetic waves via sequential absorption, reflection, and re-absorption, achieving a very small average reflection coefficient of 0.05 in the X-band. By adjusting the thickness of the MG aerogel layer to match the frequency band of interference cancelation, the absorption coefficient remains more than 0.9 in the wide frequency range of 8.2–40 GHz. Furthermore, the ODA on the 2D sheets can enhance the hydrophobicity of the aerogel. The bilayer aerogel with high porosity, adjustable conductivity, and low density (10 mg cm^−3^) is well suitable for EMI shielding, infrared stealth and camouflage, thermal insulation, solar-thermal heating, and clean-up of organic solvents and spilled crude oil.

## Experimental Section

### Materials

Octadecyl amine (ODA), toluene, and lithium fluoride (LiF, 99.99%) were supplied by Aladdin Reagents. Ti_3_AlC_2_ (400 mesh) was obtained from Jilin Yiyi Tech. Sulfuric acid (H_2_SO_4_, 98%), hydrochloric acid (HCl, 37%), hydrogen peroxide (H_2_O_2_, 30%), sodium nitrate (NaNO_3_, 98%), and potassium permanganate (KMnO_4_, 99.5%) were acquired from the Beijing Chemical Reagents. Graphite flakes were supplied by East China Graphite (China).

### Preparation of Graphene Oxide and Ti_3_C_2_T_x_ MXene Sheets

Graphene oxide (GO) was synthesized with the modified Hummers’ method [31], and an aqueous suspension of GO was prepared with the GO content of 7.5 mg mL^−1^. Ti_3_C_2_T_x_ MXene sheets were obtained by selectively etching the Al layer of the Ti_3_AlC_2_ MAX phase with a HCl/LiF etching solution (Fig. S1). After 100 mL of a HCl solution (9 M) was added into a polytetrafluoroethylene (PTFE) reactor in an ice-water bath, 8 g of LiF was added and dissolved, generating the HCl/LiF solution. Subsequently, 5 g of Ti_3_AlC_2_ MAX phase was added slowly into the HCl/LiF solution under stirring in an ice-water bath, and the mixture was heated to 35 °C for 42 h. The resultant product was washed with deionized water and centrifuged at 3500 r min^−1^ until the filtrate reached a pH value of ~ 6. The washed product was ultrasonically exfoliated in an argon atmosphere, centrifuged at 3500 r min^−1^ for 60 min to isolate the upper dispersion, and concentrated at 8000 r min^−1^ to obtain MXene sheets. The MXene and GO sheets exhibit average sizes of ~ 756 and ~ 645 nm, respectively (Fig. S2).

### Preparation of the MG Emulsion Ink and the MXene Emulsion Ink

The mixture of the MXene and GO suspensions was added into a toluene solution of ODA (15 mg mL^−1^) and emulsified using a homogenizer at 1500–3000 r min^−1^ for 10 min, generating a MXene/GO (MG) emulsion ink with a water phase volume fraction of 0.6–0.71, which was designated as M*x*G*y* ink, where the numbers of *x* and* y* represent the volume proportions of the MXene suspension and the GO suspension in the aqueous phase, respectively. The corresponding ink compositions are listed in Table S1. Similarly, an MXene emulsion ink was obtained by homogenizing the mixture of the MXene suspension and the toluene solution of ODA (15 mg mL^−1^).

### 3D Printing and Molding of Aerogels

A 3D model was created using AutoCAD and imported into an Adventure-Technology double-needle printer. The MXene emulsion ink was loaded into the 410 μm nozzle 1, while the MG emulsion ink was loaded into the 410 μm nozzle 2. The MG layer was printed first, and the MXene layer was subsequently printed on the MG layer. The resultant bilayer sample was frozen at − 10 °C and freeze-dried to generate a bilayer MG-MXene aerogel, which was denoted as MG*a*L-MXene*b*L, where *a* and *b* are the numbers of the printed MG aerogel layers and the MXene aerogel layers, respectively. In addition, the MXene emulsion ink and the MG emulsion ink were also poured successively into a polytetrafluoroethylene (PTFE) mold, frozen at −10 °C, and freeze-dried to yield a bilayer aerogel (MG*c*-MXene*d*), where *c* and *d* (mm) are the thicknesses of the MG aerogel layer and the MXene aerogel layer, respectively.

### Characterizations

The morphology and microstructures of Ti_3_AlC_2_ MAX phase, Ti_3_C_2_T_x_ MXene sheets, and bilayer aerogels were observed using a Hitachi S4700 field-emission scanning electron microscope (SEM) and a Hitachi 7700 transmission electron microscope (TEM). The chemical compositions of Ti_3_AlC_2_ MAX phase and MXene sheets were characterized with a Rigaku D/Max 2500 X-ray diffractometer (XRD). The MXene and MG-MXene aerogels were analyzed using a Nicolet Nexus 670 Fourier-transform infrared (FT-IR) spectrometer. Water contact angles were measured on a Krüss DSA100 contact angle tester. Interfacial tensions were evaluated with a Lauda Scientific LSA100 multifunctional tensiometer using a pendent-drop method. The droplet deformation and fluid flow were recorded with a digital camera attached to the tensiometer. The electrical resistance (*R*) was measured on a Fluke 12E Plus multimeter. The electrical conductivity (σ) was calculated using the equation: σ = *L*/(*R h d*), where *L*, *h*, and *d* represent the length, width, and thickness of the samples, respectively. EMI shielding performances were measured using a Keysight N5247A PNA series vector network analyzer (VNA) in the frequency range of 8.2–40 GHz. The EMI shielding performances of the aerogels with sizes of 22.86 × 10.16, 15.8 × 7.9, 10.7 × 4.3, and 7.1 × 3.6 mm^2^ were tested in the range of X-band (8.2–12.4 GHz), Ku-band (12.4–18 GHz), K-band (18–26.5 GHz), and Ka-band (26.5–40 GHz), respectively. Electrothermal temperatures of the aerogels were measured with a FLIR-H16 infrared camera. The rheological properties of the emulsions were analyzed on an Anton Paar MCR302 rheometer. A CEL-HX UV300 xenon lamp was used to provide simulated solar light. 0.5 mL droplets of used pump oil were dripped onto the aerogels to record the adsorption times. The aerogels were placed in the mixture of 3 mL Sudan Red-dyed cyclohexane and 20 mL pure water to evaluate the spontaneous oil–water separation efficiency. Similarly, 0.2 mL crude oil droplets were dripped onto the aerogels and the adsorption times were recorded with and without the downward solar light irradiation. Thermal conductivities were measured on a Hot Disk TPS 2500S thermal conductivity meter according to the ISO 22007–2 standard.

## Results and Discussion

### Design of Multifunctional Asymmetric Bilayer MG-MXene Aerogel

**Fig. 1:**
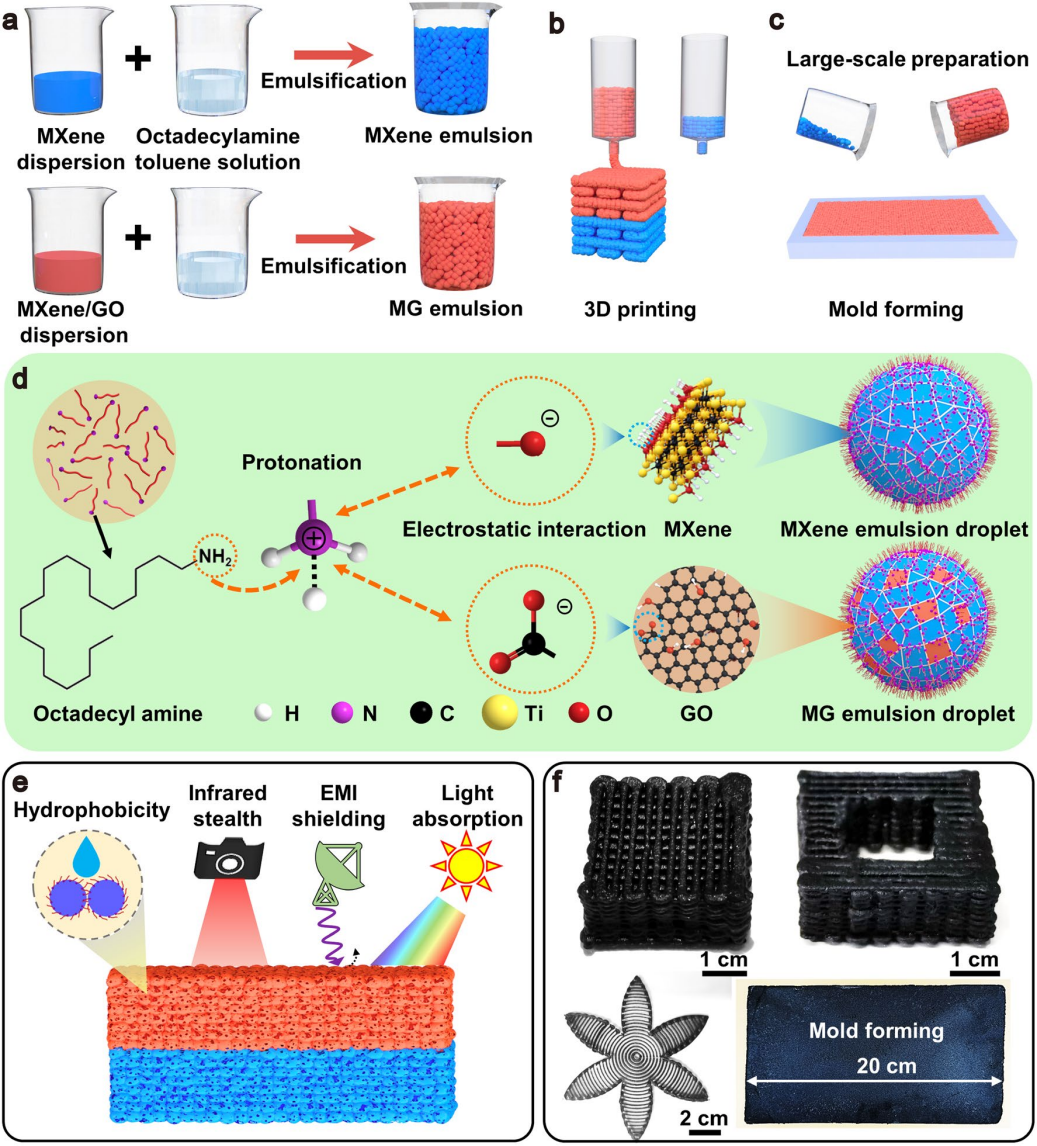
Schematic illustrating **a** preparation of MXene and MG emulsion inks, **b** 3D printing and **c** molding of bilayer MG-MXene aerogels. **d** Interfacial assembly mechanisms of MXene and MG emulsions. **e** Schematic illustrating multi-functionalities of the bilayer aerogels. **f** Digital photographs of 3D-printed aerogels with customizable shapes of petal, hollowed cube and lattice scaffold, and a large-scale aerogel by molding

Electromagnetic shielding materials are widely used in electromagnetic protection, but they usually face impedance mismatch resulting in reflection of electromagnetic wave, which aggravates electromagnetic pollution. Therefore, we designed the multifunctional asymmetric bilayer MG-MXene aerogel with ultrahigh absorption-dominated EMI shielding, infrared camouflage, solar-thermal heating, and clean-up of organic solvents and spilled crude oils, which is developed to solve increasingly serious electromagnetic radiation and pollution and meet multi-scenario applications [1, 32]. Figure 1 schematically illustrates the preparation and interfacial assembly mechanisms of the MXene and MG emulsion inks, and the construction of multifunctional bilayer aerogels by 3D printing or a large-scale molding process. The formation of stable emulsion inks is based on the electrostatic assembly of the aqueous phase of MXene and GO sheets with the organic phase of ODA at the O/W interfaces (Fig. 1a). The viscosity and stability of the emulsion inks are crucial for custom construction of 3D architectures by 3D printing (Fig. 1b) and large-scale molding (Fig. 1c).

The hydrophilic amino groups of the ODA can capture protonated hydrogen from water, generating positively charged C_18_H_37_-NH_3_^+^, which attracts negatively charged groups, such as deprotonated carboxyl (–COO^−^) and hydroxyl (–OH) groups of MXene and GO sheets, inducing the formation of nano-surfactants (Fig. 1d). These nano-surfactants accumulate and jam at the O/W interfaces, which can stabilize the shape of the aqueous droplets and prevent the aqueous droplets from fusing and aggregating [33], thus forming stable and viscous MXene and MG emulsions. After prinitng/molding and subsequent freeze-drying, the resultant bilayer aerogels integrate adjustable EMI shielding, high thermal insulation and hydrophobicity, showing significant application prospects for absorption-dominated EMI shielding, infrared stealth, Joule heating, and solar-thermal adsorption of crude oil (Fig. 1e). To meet rheological requirements for both 3D printing and molding, the rheological behavior of the emulsions is tuned by varying the oil–water interface area, which can be adjusted by the ratio of O/W phases and the stirring rate while keeping the inks concentrations unchanged. The resultant emulsion inks are suitable for custom construction of 3D architectures by 3D printing and large-area molding (Fig. 1f).

### Interface Assembly, Microstructure and Rheological Properties of Emulsions and Microstructure of Aerogels

**Fig. 2:**
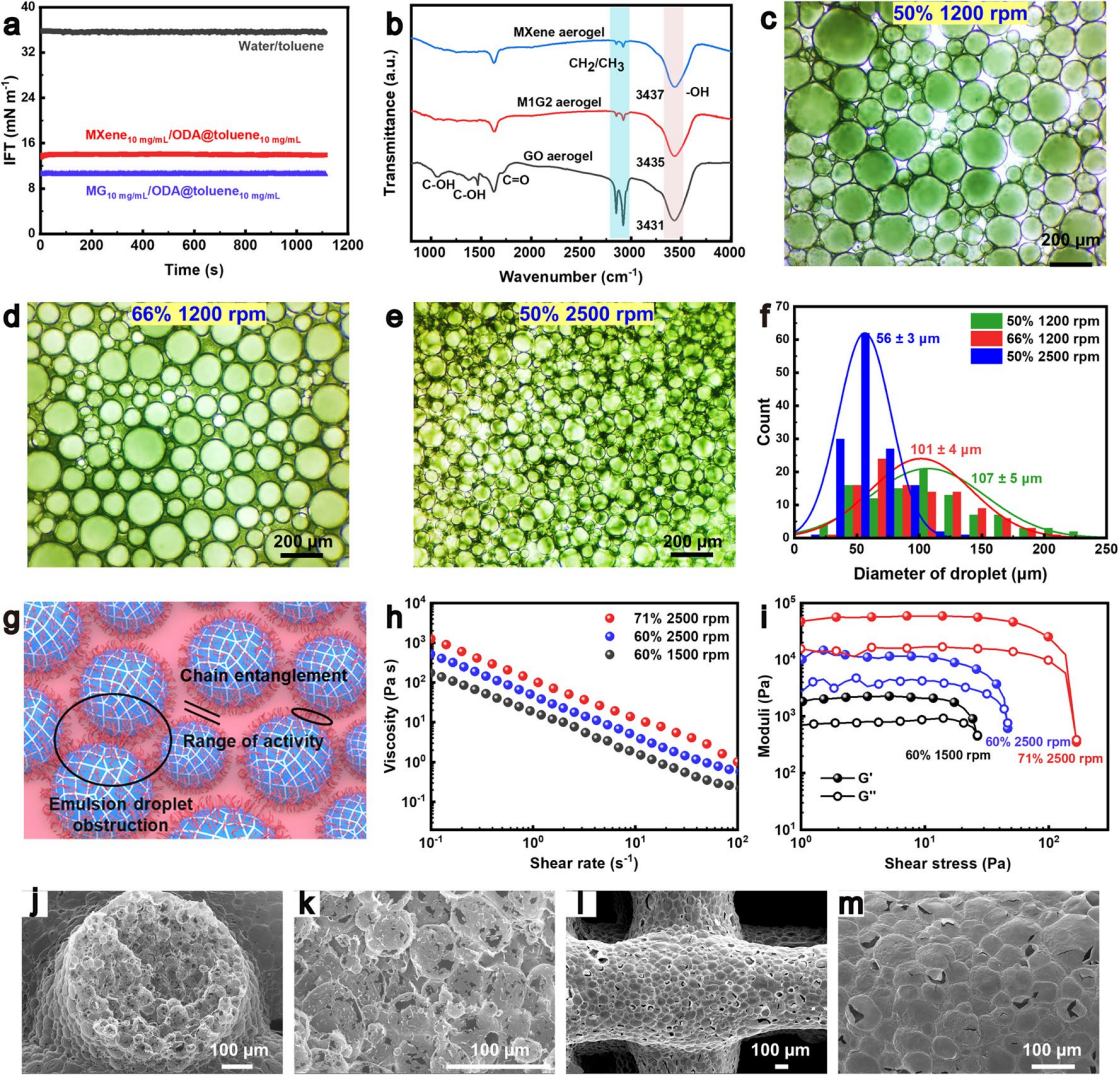
**a** Dynamic interfacial tensions of different O/W systems, including water/toluene, aqueous MXene dispersion (10 mg mL^−1^)/ODA@toluene (10 mg mL^−1^), and aqueous MG dispersion (10 mg mL^−1^, equal MXene/GO mass fractions)/ODA@toluene (10 mg mL^−1^). **b** FT-IR spectra of bilayer aerogels. **c**–**e** Optical images and **f** pore size distributions of MXene emulsions with different water phase volume ratios and stirring rates. **g** Schematic illustrating the regulation of rheological properties of the emulsion. **h** Logarithm plots of viscosity versus shear rate for the M1G2 emulsion ink. **i** Logarithm curves of storage modulus (G′) and loss modulus (G′′) versus shear stress for the M1G2 emulsion. SEM images of **j**, **k** cross-sectional and **l**, **m** top-views of printed MXene aerogel filaments

The generation of the nano-surfactants by the inorganic sheets and ODA at the O/W interface is verified by the dynamic interfacial tension measurement using a pendant drop tensiometry (Fig. 2a). Upon an aqueous drop of the dispersion of MXene or MXene/GO sheets into a toluene solution of ODA, the inorganic sheets rapidly migrate to the O/W interfaces and bond with the organic C_18_H_37_-NH_3_^+^ due to their electrostatic interactions (Figs. 2b and S3), leading to the interfacial tensions of 13.9 and 10.1 mN m^−1^, respectively, smaller than that of neat water and toluene (35.5 mN m^−1^). The pendent droplet shows a crumpled shape in the extraction process, demonstrating the formation of an interfacial film as the nano-surfactant (Fig. S4).

The rheological behavior of the emulsion inks is regulated by varying the stirring rate and the ratio of the oil/water phases. When the volume fraction of the water phase is constant, the higher stirring rate decreases the size of the emulsion droplets with a more uniform size distribution (Fig. 2c, e, f). The faster stirring results in more O/W interfaces, thereby enlarging the surface area of the interface. Conversely, when the stirring rate is constant, increasing the volume fraction of the water phase leads to a rise in the number of emulsion droplets and a slight decrease in their size (Fig. 2c, d, f). Generally, the rheological behavior of an emulsion ink is influenced by its internal friction, which is determined by the interaction and entanglement of the oleophilic chains in the nano-surfactants, the impediment to the droplet motion, and the relative movement range of the droplets (Fig. 2g) [34].

The increased interface area enhances the molecular entanglement of the oleophilic chains in the nano-surfactants, thereby increasing the internal friction of the system. Additionally, the higher number of emulsion droplets increases physical contact possibility of the droplets with each other, further obstructing droplet motion and raising the internal friction force of the system. These mechanisms elevate the friction within the emulsion ink, thereby enhancing its viscoelasticity. Thus, without altering the concentrations of MXene, GO, and ODA, or adding rheological regulators, the rheological properties of the emulsion ink can be easily regulated by adjusting the stirring rate and the volume fraction of the water phase. For instance, the increase in stirring rate from 1500 to 2500 r min^−1^ markedly enhances the viscosity (from 177 to 1252 Pa s at a shear rate of 0.1 s^−1^) and viscoelasticity (G′ from 2141 to 10,967 Pa) of the MG emulsion ink with a water phase volume fraction of 60%. The viscosity and G′ come to 931 Pa s (at a shear rate of 0.1 s^−1^) and 60,245 Pa for the MG ink with a water phase volume fraction of 71%, respectively (Fig. 2h, i). The rheological properties of the emulsion ink, including high viscosity of > 10^2^ Pa s, distinct shear-thinning behavior, and high G′ of > 100 Pa, meet the 3D printing requirement [21, 35].

The SEM images show that the 3D-printed MXene and MG aerogels consist of uniform filaments with nearly circular cross-section contours and welded filament intersections (Fig. 2j, l, and S5). The self-supporting capability of the aerogels demonstrates the suitability of the emulsion inks for 3D printing. The filament possesses closely packed spherical pores with close-cell structure with diameters of ~ 46 μm (Fig. S6). Notably, although the formed nano-surfactant film can sustain its original structure without collapse during the freeze-drying process, the growth and sublimation of ice crystals puncture the nano-surfactant films, leaving secondary holes on the pore walls of the numerous close-cells (Figs. 2k, m and S5d, h, l). Consequently, the multi-scale porous structure would benefit the integration of multifunctional and ultra-lightweight characteristics of the aerogels.

### EMI Shielding Performances

Bilayer MG-MXene aerogels are constructed by using a dual-needle 3D printing technology for EMI shielding with ultra-high absorption. The MG layer as the absorption layer can optimize the impedance match and attenuate incident electromagnetic waves by dielectric loss [36]. Meanwhile, the MXene layer acts as an effective reflection layer for preventing the transmission of the remaining electromagnetic waves.

**Fig. 3:**
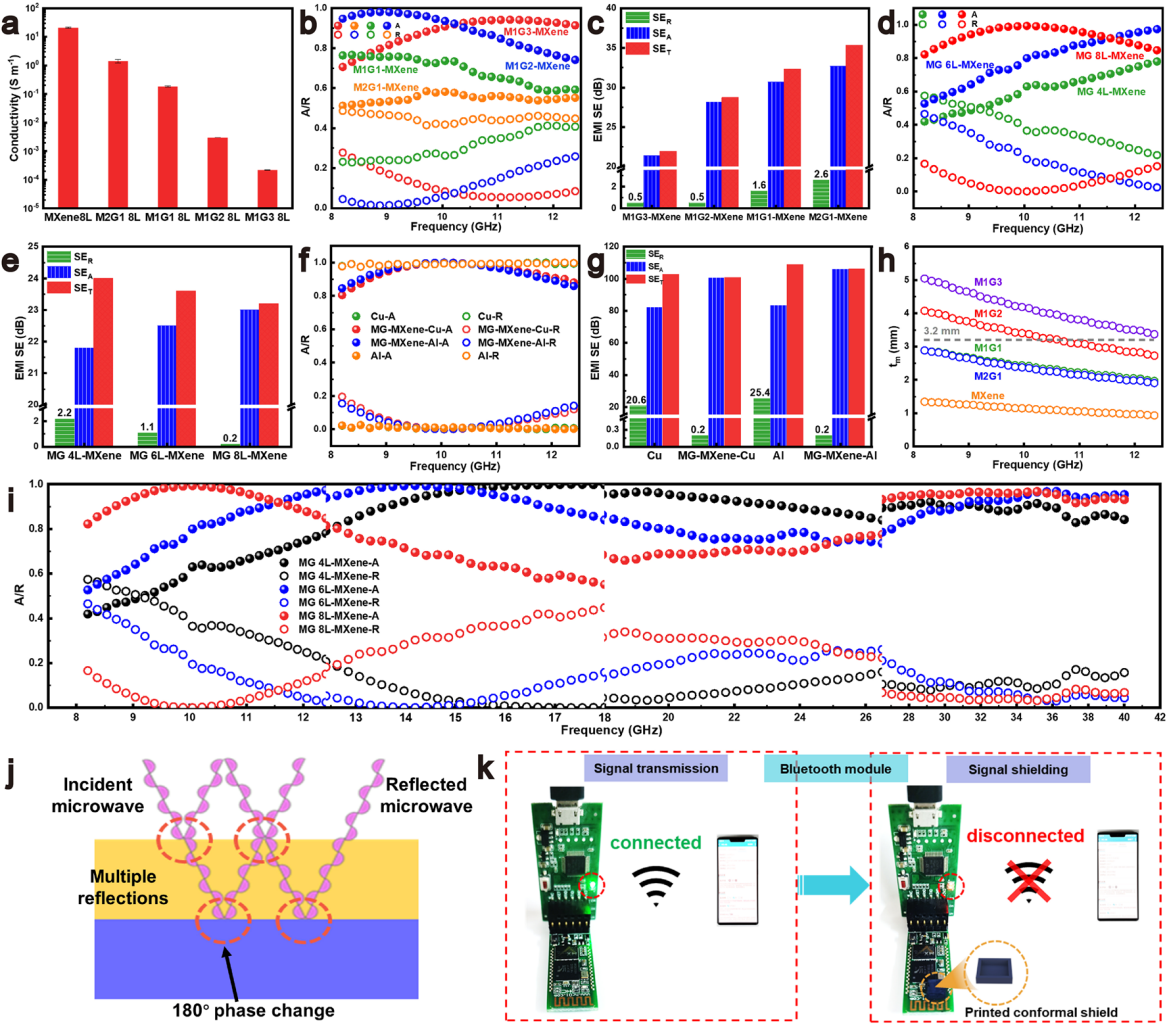
**a** Conductivities of MXene 8L and MG 8L with different GO contents. Plots of **b** absorption and reflection coefficients, and **c** SE_A_, SE_R,_ and SE_T_ of MG 8L-MXene 8L with different GO contents. Plots of **d** absorption and reflection coefficients, and **e** SE_A_, SE_R_, SE_T_ of M1G2-MXene 6L with different M1G2 aerogel thicknesses. Plots of **f** absorption and reflection coefficients, and **g** SE_A_, SE_R_, SE_T_ of M1G2 8L-MXene 6L-Cu and M1G2 8L-MXene 6L-Al. **h** Dependences of matching thickness (*t*_*m*_) on the matching frequency at a wavelength of λ/4. **i** Absorption and reflection coefficients of M1G2-MXene 6L with different M1G2 layers. **j** Schematic of the extinction effect at air-MG and MG-MXene interfaces. **k** Display of signal shielding of Bluetooth module by a 3D-printed EM shielding cover

To achieve satisfactory absorption and reflection of the MG and MXene layers, the MXene/GO ratio in the aqueous phase is tuned. Typically, increasing the GO concentration significantly reduces the electrical conductivity of the MG aerogel (Figs. 3a and S7), along with decreased real and imaginary part of the complex permittivity (Fig. S8a, b) and declined dielectric tangential loss (Fig. S8c). These changes signify a weakened dielectric loss capacity but an improved impedance matching (Fig. S8d). We further explore the effects of the GO content in the MG layer on the EMI shielding efficiency. Specifically, increasing the GO content in the MG layer optimizes the impedance match and enhances the absorption coefficient of the MG-MXene aerogel (Fig. 3b). Impressively, the average absorption coefficient in the X band increases from 0.55 for M2G1 to 0.89 for M1G2 (Fig. S9), indicating an absorption-dominant EMI shielding mechanism. The M1G3/MXene and M1G2/MXene bilayer aerogels exhibit almost the same SE_R_ of 0.5 dB, but the SE_T_ values reduce from 28.7 dB of M1G2 to 21.9 dB of M1G3 (Fig. 3c), confirming that the further increase in the GO content decreases the absorption and increases the transmission of the electromagnetic waves, ultimately reducing the overall EMI shielding performances.

The thicknesses of the MG and MXene layers, determined by the number of printed layers, play a pivotal role in the modulation of the interaction between aerogels and electromagnetic wave. When the thickness of the MG layer increases from 4 to 8 layers, the absorption coefficient of the MG-MXene aerogel shows an increasing trend (Fig. 3d) while SE_R_ reduces from 2.2 dB of MG 4L-MXene, to 1.1 dB of MG 6L-MXene and 0.2 dB of MG 8L-MXene (Fig. 3e). The negligible SE_R_ values indicate that majority of the electromagnetic waves are absorbed by the MG layer, leading to an ultrahigh absorption coefficient value of up to 0.95 in the X-band. Therefore, increasing the thickness of the MG aerogel improves absorption and reduces reflection of the electromagnetic waves. Increasing the thickness of the MXene layer is beneficial to the EMI shielding efficiency of the bilayer MG-MXene aerogel with minimum influence on its ultrahigh electromagnetic wave absorption feature. With an 8-layer MG layer and an incremental increase in the MXene layer from 4 to 8 layers, the SE_T_ increases from 18 dB of MG-MXene 4L to 23.2 dB of MG-MXene 6L, and further to 32.5 dB of MG-MXene 8L (Fig. S10). Therefore, the MXene layer plays dual roles of actively absorbing electromagnetic energy and simultaneously enhancing the re-absorption process inside the bilayer structure.

To modify its reflection and absorption ability, the MG-MXene aerogel is attached to highly conductive copper or aluminum metal foils. Normally, when an incident electromagnetic wave reaches the metal surface, the high conductivity of the metal causes an impedance mismatch, leading to substantial electromagnetic wave reflection [37], with the reflection coefficient of closing to 1 (Fig. 3f). However, the M1G2 8L-MXene6L aerogel with the metal foils significantly reduce the reflection coefficient to 0.05, demonstrating a dramatic improvement in mitigating the inherent reflective properties of metals (Fig. S11). The absorption coefficients of MG and MXene aerogels printed on aluminum foils are 0.57 and 0.41 and their SE_R_ values are 2.5 and 3.9 dB, respectively (Fig. S12), verifying the contribution of the MXene aerogel to the electromagnetic wave absorption and the effect of MG-MXene structure synergy on electromagnetic wave absorption. The M1G2 8L-MXene 6L aerogel maintains the extraordinary EMI shielding efficiency of copper foil (> 100 dB) with an ultralow SE_R_ of 0.2 dB (Fig. 3g). Hence, the metal foil is placed on the backside of the asymmetric bilayer aerogel, acting as a reflective backing layer. When electromagnetic waves enter the structure from the MG side (top layer), the MG layer facilitates impedance matching and initial wave absorption via multiple scattering and dielectric loss. The transmitted portion of the wave then reaches the MXene layer, attenuating the energy further via conductive and interfacial losses. Any residual unabsorbed electromagnetic waves that reach the metallic foil are reflected back into the aerogel structure, particularly toward the MXene and MG layers, where they undergo secondary absorption (re-absorption). This multiple internal reflection mechanism significantly enhances the overall absorption efficiency. These metrics showcase the capability of the MG-MXene aerogel to preserve the inherent electromagnetic wave shielding properties of metals while significantly reducing the electromagnetic wave reflection [38–40]. When the MG-MXene aerogel is flipped with the MXene layer exposing to incident electromagnetic waves (Fig. S13a), MXene 6L-M1G2 8L-Al exhibits a high SE_R_ of 4.95 dB and a high electromagnetic wave reflection value of 0.65 (Fig. S13b, c) because of the electromagnetic wave reflection by the conductive MXene layer. Apparently, the MG layer with proper electrical conductivity is crucial for impedance matching between air and the MXene/MG aerogel, which is a prerequisite for achieving high electromagnetic wave absorption [41].

Adjusting the thickness of MG layer can enhance the EMI shielding of the bilayer MG-MXene aerogel with high electromagnetic wave absorption in the broadband and vary the absorption peaks. The reduction in the thickness of the MG layer from 8 layers to 6 and 4 layers shifts the maximum absorption peak from 10 GHz to 14.3 and 17.5 GHz, respectively (Fig. 3h, i), which can be explained by the λ/4 model expressed as Eq. (1) [42]:

1 $$t_{m} = \frac{n\lambda }{4} = \frac{ nc}{{ 4f_{{m\sqrt {\left| {\varepsilon_{r} } \right|\left| {\mu_{r} } \right|} }} }} (n = 1,3,5\cdot\cdot\cdot)$$

where *t*_m_ is the matching thickness, *f*_m_ is the frequency of the maximum absorption, and *c* is the light speed in the free space. |*ε*_*r*_| and |*μ*_*r*_| represent the moduli of complex permittivity and permeability, respectively. The matching thickness of the M1G2 8L-MXene corresponds to a matching frequency of 10.5 GHz (Fig. 3h), aligning with the absorption peak observed in the X-band (Fig. 3i). As the frequency increase corresponds to the decrease in matching thickness, reducing the matching thickness can generate high absorption at the high-frequency range. When the matching thickness and the frequency satisfy Eq. (1), the extinction effect of electromagnetic waves occurs due to the phase flipping of the reflected waves between air/MG and MG/MXene interfaces (Fig. 3j) [43]. The reflected electromagnetic waves at the air/MG interface and the MG/MXene interface can result in destructive interference, enhancing the absorption of the bilayer MG-MXene aerogel. The correlation of the absorption coefficient of the MG-MXene aerogel with the MG layer thickness is corroborated by the simulation results (Fig. S14). The MG 6L-MXene aerogel achieves an average absorption coefficient of 0.95 in the range of 12.4–18 GHz, and the MG 4L-MXene aerogel absorbs over 99% of electromagnetic waves in the range of 5.9–18 GHz. By tuning the thickness of the MG aerogel layer, the absorption coefficient of more than 0.9 in the broad frequency range of 8.2–40 GHz is achieved (Fig. S15). To demonstrate a practical EMI shielding application, a 3D-printed square shielding cover (5 × 5 × 3 mm^3^) has its capability in attenuating the signal of a Bluetooth module (Fig. 3k), demonstrating the significant potential of 3D-printing in the domain of miniaturized, conformable, and customizable electronic packaging shielding [44–46].

### Finite Element Simulation and EMI Shielding Mechanisms

To elucidate the interaction between the multiscale pore structure of the MG-MXene aerogel and the incident electromagnetic waves, finite element simulations are conducted by focusing on the electric field distribution, and absorption and reflection coefficients of the MG-MXene bilayer aerogels with different structural configurations of dense, straight pore, and spherical pore structures. In the configurations devoid of pores, the electric field distribution in the M1G2-MXene compact arrangement reveals a concentration of the electric field within the M1G2 layer, rather than in the MXene layer (Fig. S16a, b). This distribution pattern indicates that the electromagnetic wave attenuation primarily occurs within the M1G2 layer, while the MXene layer blocks the electromagnetic wave transmission. Moreover, the absorption coefficients of dense M1G2-MXene layers exceed their reflection coefficients (Fig. S16c, d), showing an absorption-dominant EMI shielding performance.

**Fig. 4:**
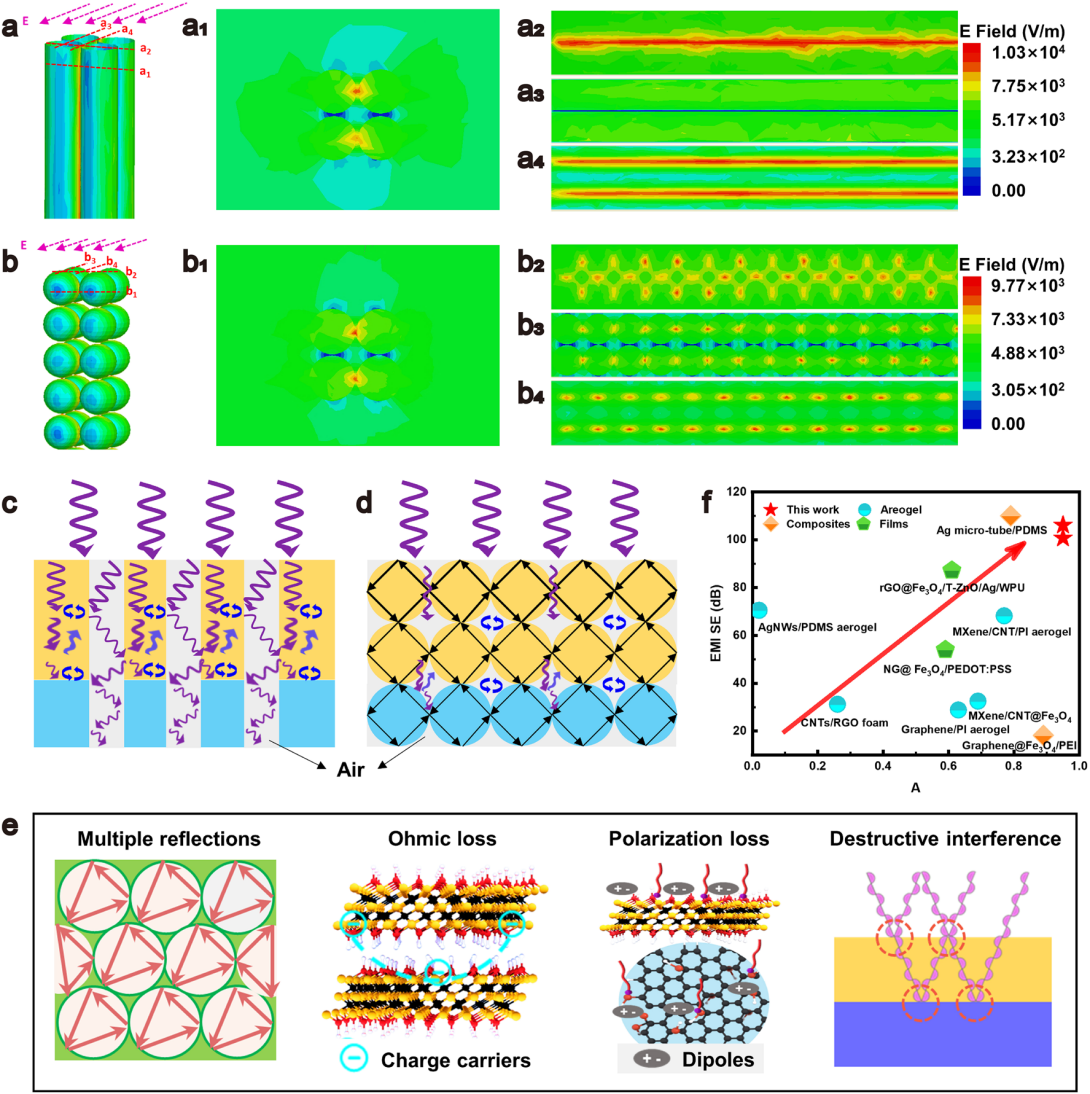
Simulated electric field distributions for **a** whole and **a**_**1**_**-a**_**4**_ vertical dashed plane of MG-MXene aerogel with straight pores, and **b** whole and **b**_**1**_**-b**_**4**_ vertical dashed plane of MG-MXene with spherical pores. Schematic illustrating electromagnetic wave attenuation in **c** straight pore structure, and **d** spherical pore structure. **e** Comparison of EMI shielding efficiency and absorption coefficient with those of EMI shielding materials reported. **f** EMI shielding mechanisms of MG-MXene aerogel

As presented in Fig. 4a, b, the introduction of straight and spherical pore structures with detailed parameters in Fig. S17 facilitates the penetration of the electromagnetic waves, resulting in pronounced electric field distributions within the pores (Fig. 4a1, b1). The pore interfaces parallel to the electric field direction exhibit strong electric field distribution (Fig. 4a2–a4, b2–b4) and substantial ohmic loss. Notably, the electric field distribution associated with the spherical pore structure demonstrates a more pronounced attenuation as compared to the straight pore structure (Fig. S18). Furthermore, electric field intensity distribution along the pore interfaces of MG-MXene is illustrated (Figs. S19–S21). The spherical pore structure has rich interfaces in and between its pores, which is conducive to the strong ohmic loss of the electromagnetic waves at the interface and the reflection and scattering of the electromagnetic waves, showing outstanding absorption capability as compared with the porous structures formed by random-freezing and directional-freezing (Fig. S22).

The EMI shielding mechanisms of the bilayer MG-MXene aerogel are depicted in Fig. 4c–e. The MG layer ensures optimal impedance matching for facilitating the electromagnetic waves to enter the aerogel, thereby reducing the reflection. Due to the lower impedance of the air within the pores, the electromagnetic waves penetrate into these pores and undergo multiple reflection and attenuation at the pore interfaces [47]. Compared to the straight pore structure, the spherical closed-pore configuration offers a greater number of reflective interfaces, elongating the electromagnetic wave propagation path and enhancing the electromagnetic wave attenuation [5, 48]. Additionally, the electric field strength along the pore interfaces, parallel to the electric field direction, is intensified, resulting in pronounced ohmic loss [49–51]. The electromagnetic waves reflected at the MXene layer interfaces can be absorbed by the MG layer, ensuring a high EMI shielding performance. The high carrier density of MXene and alongside surface functional groups and defects of both MXene and GO in the MG-MXene aerogel amplify the dipole polarization loss and conductive loss, benefiting the electromagnetic wave attenuation [52–54]. The electromagnetic wave absorption of the MG-MXene aerogel can be enhanced by optimizing the thickness of the MG layer to realize the extinction effect. Apparently, the bilayer MG-MXene aerogel is among the best EMI shielding composites reported. More impressively, its average absorption coefficient still reaches 0.95 in the X band (Fig. 4f, Table S2).

### Solar-Thermal Energy Conversion Performances

**Fig. 5:**
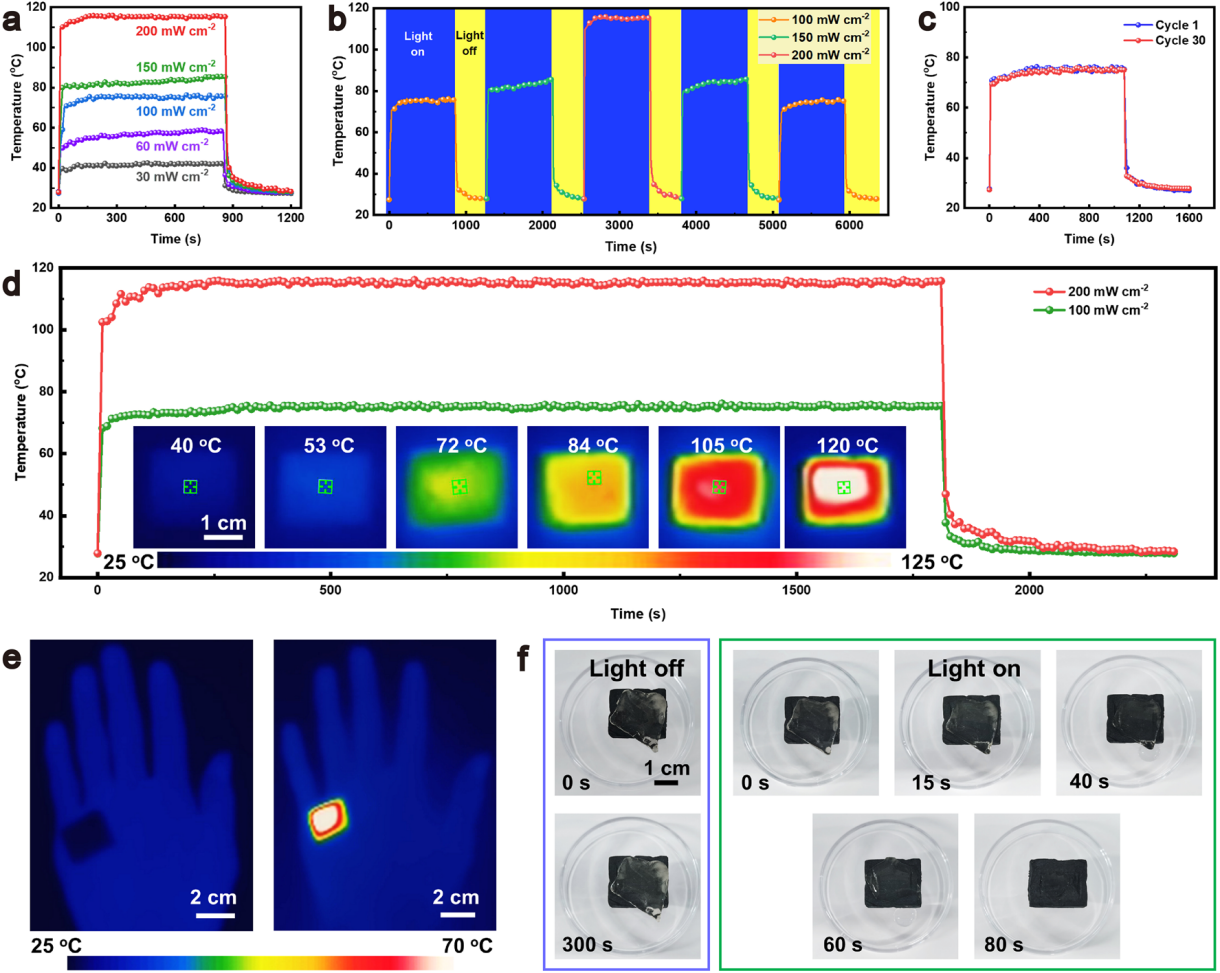
Plot of temperature versus solar radiation time of **a** MG-MXene aerogel at various light power densities, **b** MG-MXene aerogel under solar radiation with stepwise altering light power densities, **c** MG-MXene aerogel before and after 30 light on/off cycles and **d** MG-MXene aerogel under 1800 s solar radiation. Insets are infrared images of the MG-MXene aerogel during long-term solar radiation. **e** Infrared images demonstration of solar-thermal therapy. **f** Digital photographs of MG-MXene aerogel melting ice under solar radiation

In addition to the ultrahigh electromagnetic wave absorption, the MG-MXene aerogel exhibits exceptional solar-thermal energy conversion efficiency because of the local surface plasmon resonance characteristics inherent in the MXene sheets [55]. Under downward irradiation with light power intensities of 30 and 200 mW cm^−2^, the MXene surface of the MG-MXene aerogel exhibits equilibrium temperatures of 42 and 115 °C, respectively (Fig. 5a). Meanwhile, the MG surface shows the same solar-thermal conversion capability (Fig. S23). The surface equilibrium temperature can be rapidly tuned by varying the light power intensity (Fig. 5b), exhibiting fast heating and cooling responses. Furthermore, the aerogel shows stable and repeatable temperature–time curves during 30 light on/off cycles (Fig. 5c). Even under continuous irradiation at 100 and 200 mW cm^−2^ for 30 min, the aerogel maintains stable and uniform surface temperatures (Fig. 5d). The aerogel can also be used for localized heat therapy on human skins or joints on the basis of its solar-thermal energy conversion and thermal insulation effects (Fig. 5e). The efficient solar-thermal energy conversion capability of the MXene aerogel facilitates de-icing. In the absence of solar light, the ice on the aerogel melts barely after 300 s, whereas the ice can be completely melted within 80 s under the irradiation at 150 mW cm^−2^ (Fig. 5f).

### Hydrophobicity, Oil/Water Separation and Oil Absorption Capacity

**Fig. 6:**
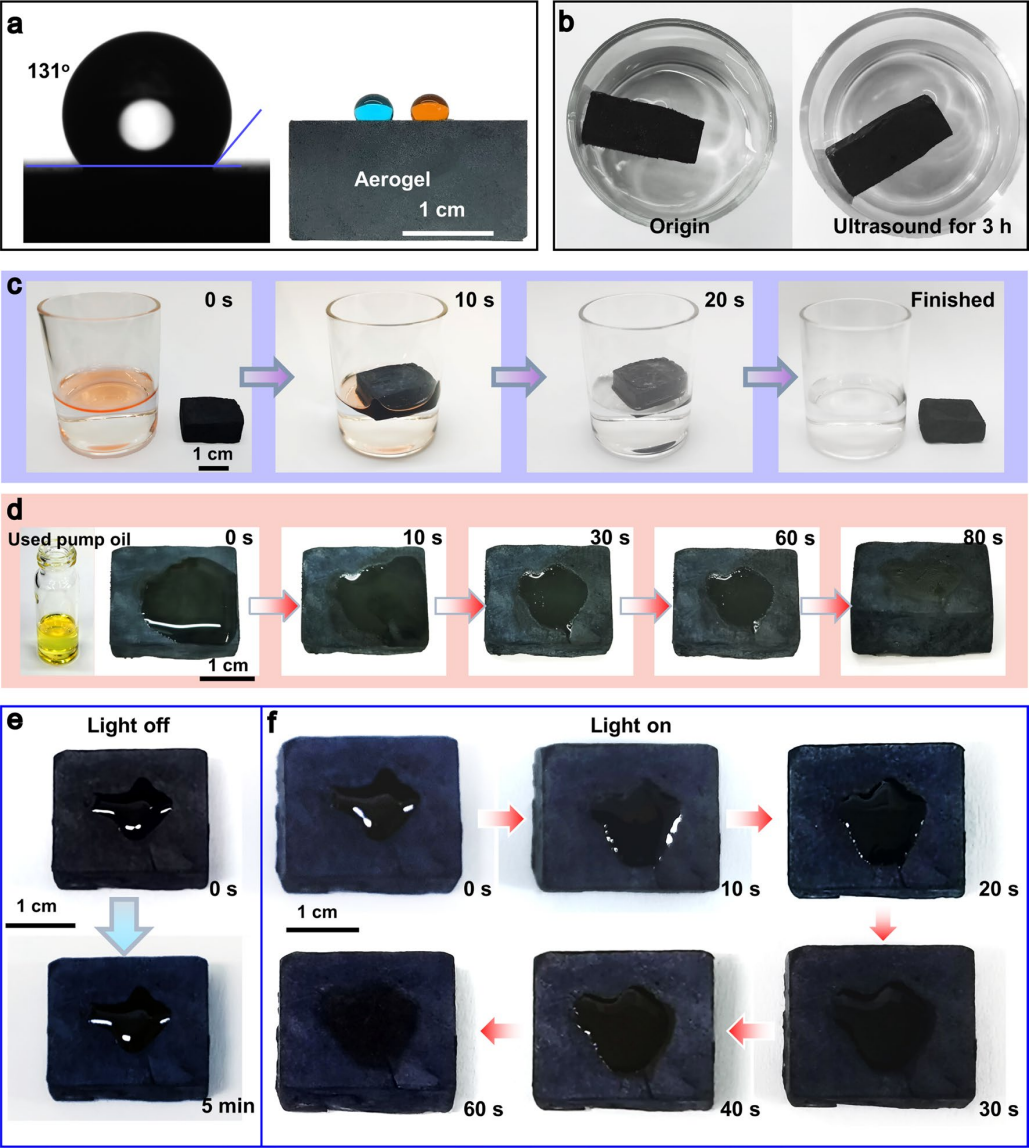
**a** Water contact angles of water on the MXene aerogel side of the MG-MXene aerogel. **b** Digital photographs of MG-MXene aerogel before and after ultrasonic treatment in water for 3 h. **c** Digital photographs of MG-MXene aerogel absorbing an organic solvent. **d** Absorption of used pump oil. Adsorption of crude oil **e** without and **f** under solar-thermal irradiation

The MG-MXene bilayer aerogel is efficient in absorbing organic solvents and oils because of its hydrophobic surface deriving from the presence of ODA on MXene and GO sheets [56]. As shown in Fig. 6a, the MXene aerogel side of the MG-MXene aerogel shows a water contact angle of 131°, proving its hydrophobicity. The resultant hydrophobic aerogel is also structurally stable demonstrated by being continuously ultrasonicated in water for 3 h (Fig. 6b). It can absorb cyclohexane in 20 s and waste oil in 80 s (Fig. 6c, d). Accelerated by the solar-thermal energy conversion ability of the black aerogel, the viscous crude oil can be completely absorbed in 60 s (Fig. 6e, f). Apparently, the MG-MXene aerogel is promising for solar-thermal collection of organic compounding and pollutants.

### Thermal Insulation and Infrared Stealth

**Fig. 7:**
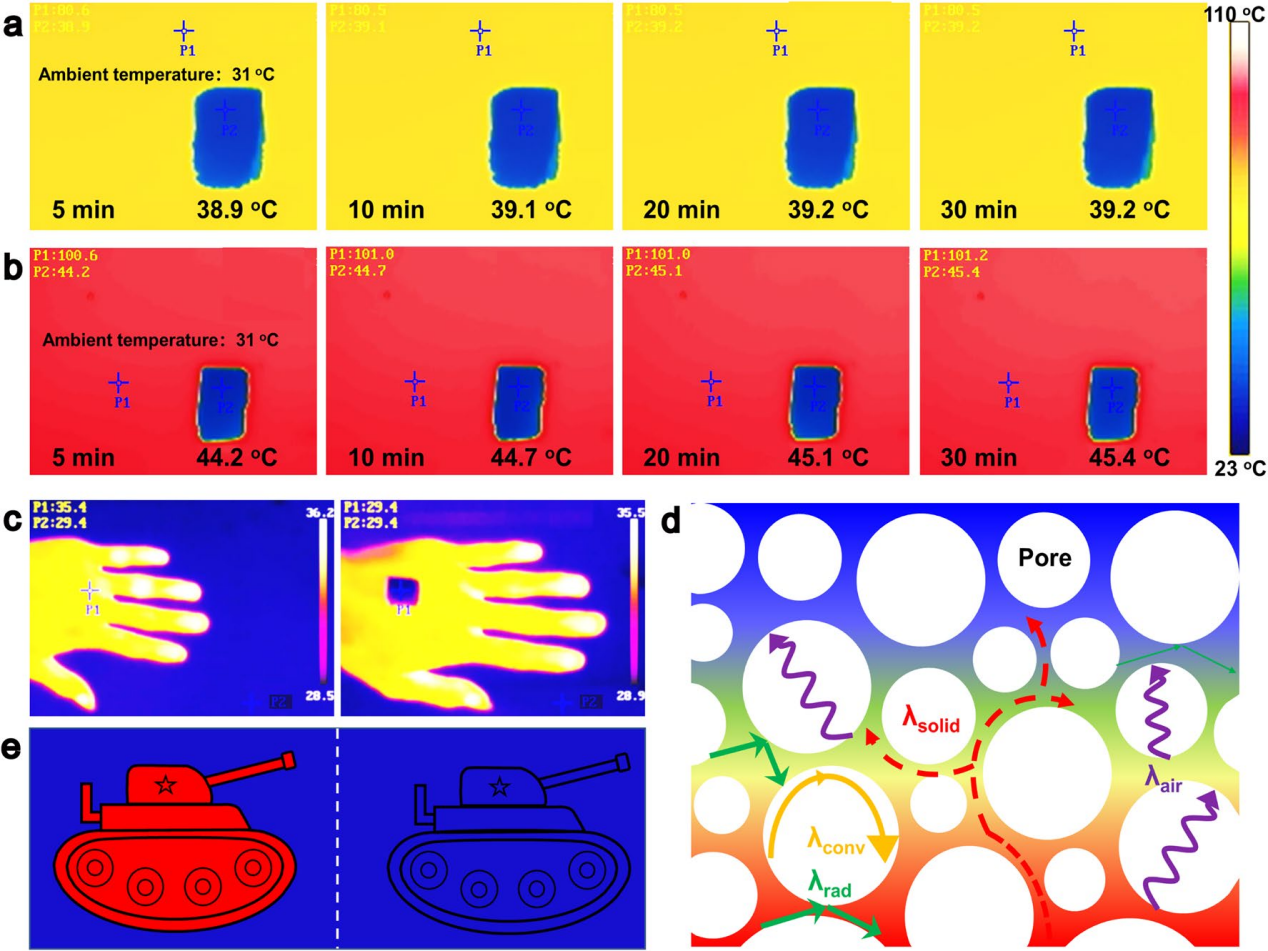
Infrared thermal imager images of M1G2-MXene aerogels placed on **a** 80 °C heating platform for different times, **b** 100 °C heating platform for different times, and **c** human hand. **d** Thermal insulation mechanism of M1G2-MXene aerogel. **e** Schematic diagram of infrared stealth

In addition, the MG-MXene bilayer aerogel possesses superior thermal insulation and infrared stealth capabilities. As shown in Fig. 7a, after placing on a heating platform of 80 °C for 30 min, the top surface of the M1G2-MXene aerogel with a thickness of 1 cm maintains a low temperature of ~ 39.2 °C, which is merely  ~ 8.2 °C higher than the ambient temperature of 31 °C (Fig. S24). Similarly, at a higher platform temperature of 100 °C, the M1G2-MXene aerogel can suppress its top surface temperature to 45.4 °C (Fig. 7b), implying a 14.4 °C difference from the ambient temperature of 31 °C (Fig. S24). These results demonstrate the excellent thermal insulation performances of the M1G2-MXene aerogel makes it suitable for shielding infrared radiation. For example, as shown in Fig. 7c, the infrared thermal image of a human hand under ambient conditions presents a temperature of 35.4 °C, distinct from its surrounding of 29.4 °C. However, when covering with the M1G2-MXene aerogel, in the infrared thermal imager, the covered area assimilates into the environmental backdrop, achieving effective infrared camouflage.

The thermal insulation mechanism of the M1G2-MXene aerogel is illustrated in Fig. 7d. Theoretically, the thermal conductivity (*λ*) of the aerogel in air is given by $\lambda = \lambda \,{\text{conv}} + \lambda \,{\text{solid}} + \lambda \,{\text{air}} + \lambda \,{\text{rad}}$, where $\lambda {\text{conv}}$, $\lambda {\text{solid}}$, $\lambda {\text{air}}$, and $\lambda {\text{rad}}$ represent heat conduction, solid-phase thermal conductivity, gas-phase thermal conductivity, and thermal radiation, respectively [57, 58]. Since the micro/nanopore sizes are well below the critical size of 1 mm required for natural convection, $\lambda \text{conv}$ can be negligible. Due to the low infrared emissivity of MXene, $\lambda \text{rad}$ is also negligible. The aerogel with a density of 12 mg cm^−3^ has numerous pores and contains a large amount of air inside, exhibiting a low thermal conductivity of 32 mW m^−1^ K^−1^. The low thermal conductivity and the internal spherical pore structure can inhibit the radiation heat transfer, making the aerogel possible for infrared camouflage applications (Fig. 7e).

### Joule Heating and Dynamic Infrared Camouflage

**Fig. 8:**
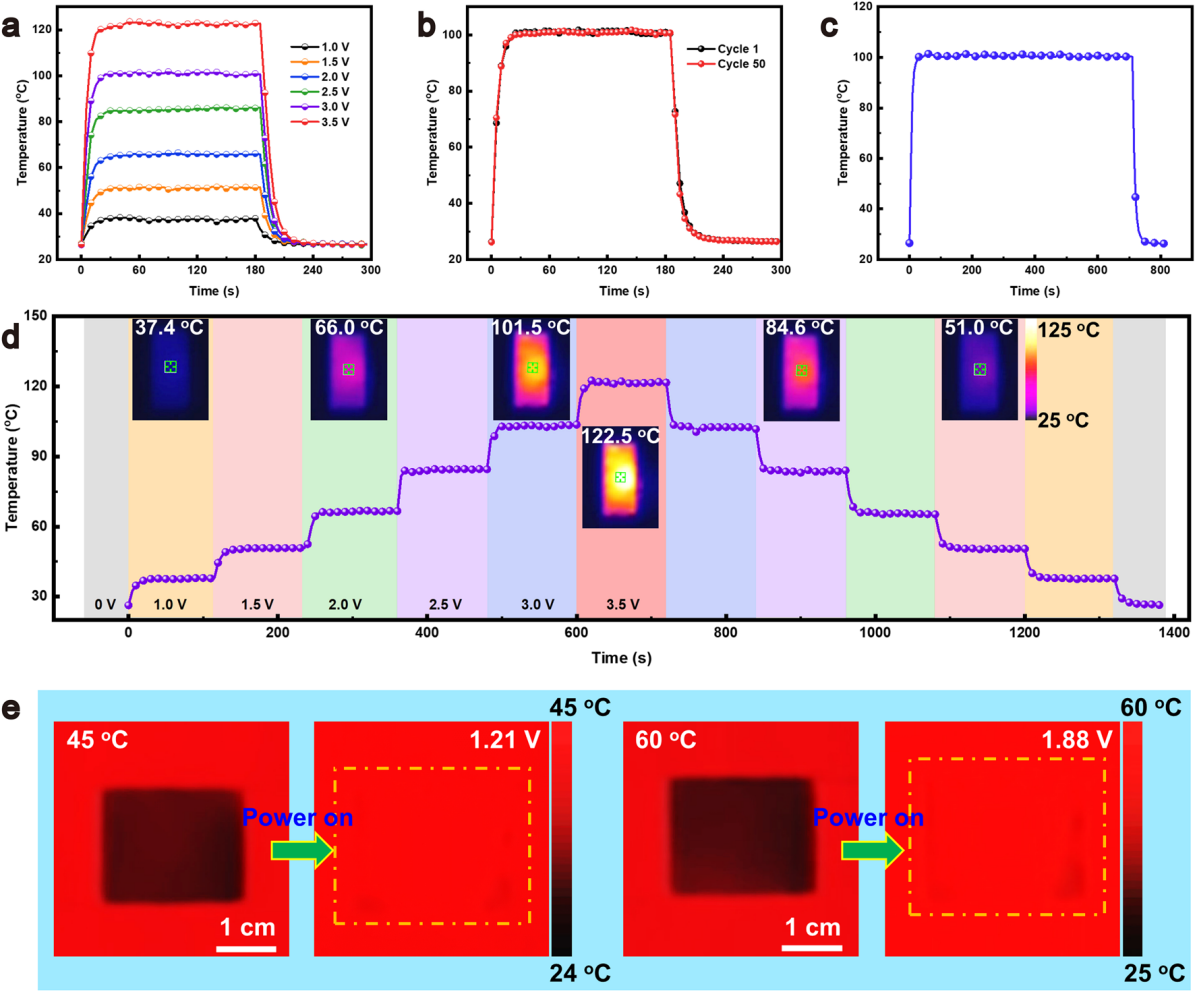
**a** Surface temperatures of the MXene aerogel side of the bilayer aerogel at different voltages. **b** First-cycle and 50th-cycle temperature–time curves of the MXene aerogel side of the bilayer aerogel. **c** Long-term Joule-heating stability. **d** Cyclic stability of gradient voltage rise and fall and infrared images at different voltages. **e** Demonstration of dynamic infrared camouflage

Furthermore, the aerogels are capable of achieving widely adjustable and stable surface temperatures via Joule heating. Increasing the voltage from 1.0 to 3.5 V with an interval of 0.5 V results in respective rises in equilibrium temperature from 37.8 to 122 °C (Fig. 8a). After 50-cycle Joule heating at 3 V, the stable surface temperatures of the aerogel demonstrate its remarkable cyclic stability and repeatability (Fig. 8b). Even extending the Joule heating over 700 s, the aerogel still maintains a stable surface equilibrium temperature (Fig. 8c), indicating its excellent long-term operational stability. The rapid heating and cooling capabilities of the aerogel enable quick surface temperature adjustments in response to voltage changes, ensuring a uniform surface temperature in the power-on state observed by an infrared camera (Fig. 8d), confirming its cyclic heating/cooling stability [59]. The stable, repeatable, and enduring Joule heating performance and the rapidly adjustable surface temperatures make the aerogels suitable for dynamic infrared camouflage applications. In an environment of 45 °C, the superior thermal insulation of the aerogel results in a lower surface temperature within the field of view of an infrared camera. Upon applying a voltage of 1.21 V, the aerogel merges into surrounding environment under an infrared detection (Fig. 8e), achieving dynamic infrared camouflage and evading infrared detection. By adjusting the voltage, the infrared camouflage can be attained across various environmental temperature backgrounds. Utilizing Joule heating to achieve dynamic infrared camouflage significantly expands potential applications of the aerogels in infrared stealth technology [60, 61].

## Conclusions

Electrically conductive yet thermally insulating, ultralight and hydrophobic bilayer aerogels with closed spherical pores are printed for EMI shielding with ultrahigh absorption, infrared camouflage, solar-thermal heating, and clean-up of organic solvents and spilled crude oils by using an emulsion ink derived from the interface electrostatic assembly of ODA with MXene/graphene oxide sheets and subsequent freeze-drying. The top MG aerogel layer of the bilayer aerogel acts as an incident layer of electromagnetic waves to optimize impedance matching by incorporating GO and achieve thickness matching of a quarter wavelength, while the bottom MXene aerogel layer of the bilayer aerogel suppresses electromagnetic wave transmittance. Thanks to the absorption–reflection–reabsorption mechanisms and the interference cancelation effect, the bilayer aerogel achieves ultrahigh absorption coefficient of 0.95 in the X-band. By modulating the thickness of the MG aerogel layer, the absorption coefficient in the broadband of 8.2–40 GHz exceeds 0.9. The finite element simulation results of the electric field intensity distribution in the asymmetric bilayer structure and closed-pore structure prove their ultrahigh absorption and loss capacity. Because of the adjustable rheological behavior, the emulsion ink can meet the requirements of molding and 3D printing for the preparation of large-scale and customized aerogels. Additionally, the aerogel shows application potential in electromagnetic wave absorption and shielding, dynamic infrared stealth, oil/water separation and solar-thermal management.

## Supplementary Information

Below is the link to the electronic supplementary material.

Supplementary file1 (DOCX 7553 kb)
