# Supplementary material for: Multifunctional Asymmetric Bilayer Aerogels for Highly Efficient Electromagnetic Interference Shielding with Ultrahigh Electromagnetic Wave Absorption
Source: Nanomicro Lett. 2025 Jun 12;17:291. doi: 10.1007/s40820-025-01800-6 (PMC12162446; doi:10.1007/s40820-025-01800-6)
Supplement: Supplementary file 1 — Supplementary file1 (DOCX 7553 kb) [file 40820_2025_1800_MOESM1_ESM.docx]

Supporting Information for

**Multifunctional Asymmetric Bilayer Aerogels for Highly Efficient Electromagnetic Interference Shielding with Ultrahigh Electromagnetic Wave Absorption**

Cheng-Zhang Qi^1, 2^, Peng Min^1^ *, Xinfeng Zhou^1^, Meng Jin^1^, Xia Sun^1^, Jianjun Wu^3^, Yanjun Liu^3^, Hao-Bin Zhang^1^ *, Zhong-Zhen Yu^2^ *

^1^ State Key Laboratory of Organic-Inorganic Composites, Beijing University of Chemical Technology, Beijing 100029, China

^2^ Center for Nanomaterials and Nanocomposites, College of Materials Science and Engineering, Beijing University of Chemical Technology, Beijing 100029, China

^3^ Ningxiamogong Technology Co., Ltd, Yongning 750100, China

*E-mails: [pmin@buct.edu.cn](mailto:pmin@buct.edu.cn) (Peng Min); [zhanghaobin@buct.edu.cn](mailto:zhanghaobin@buct.edu.cn) (Hao-Bin Zhang); [yuzz@mail.buct.edu.cn](mailto:yuzz@mail.buct.edu.cn) (Zhong-Zhen Yu)

**Supplementary Figures and Tables**


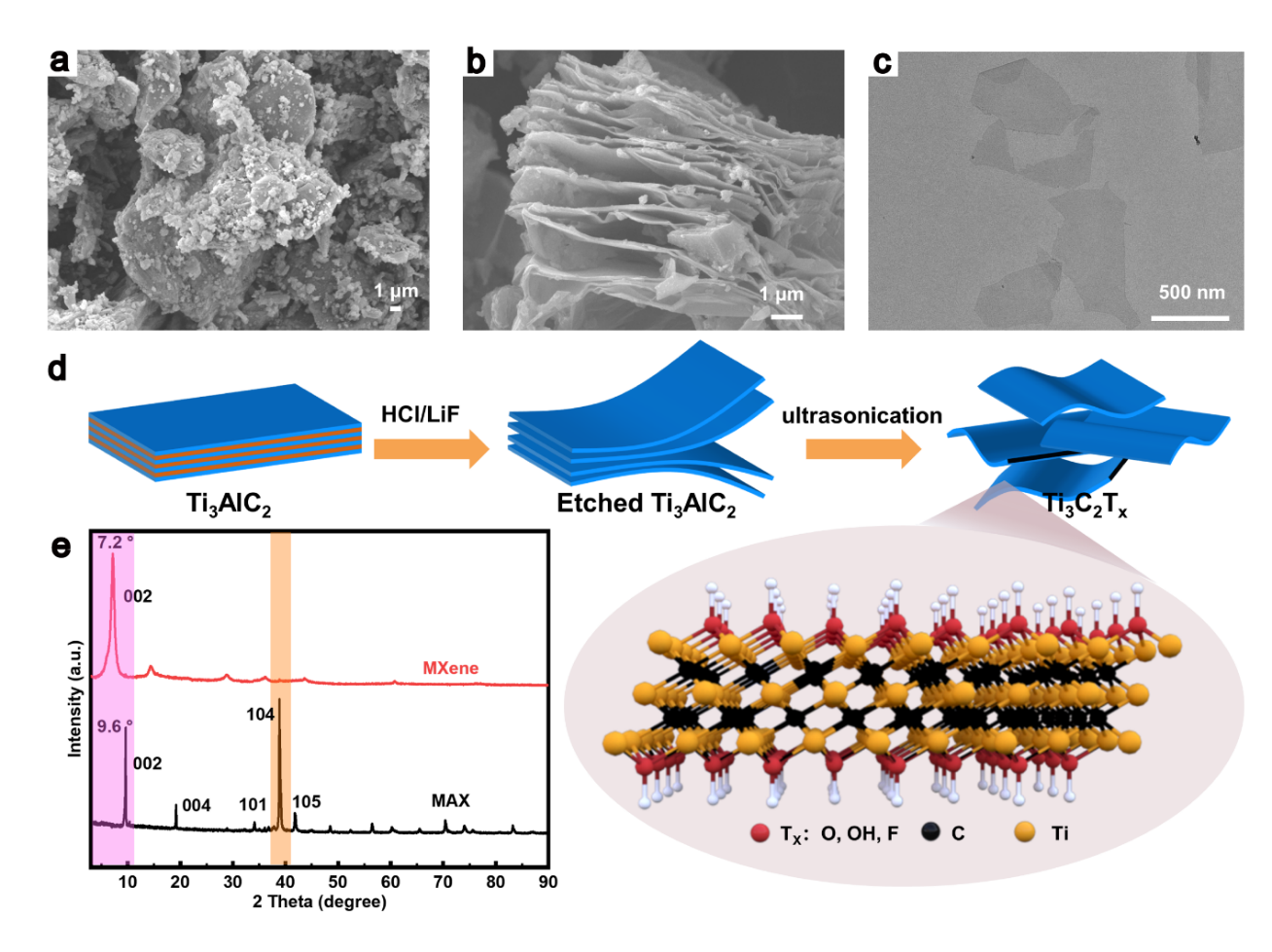


**Fig. S1** **a** SEM image of Ti_3_AlC_2_ MAX phase. **b** SEM image of etching phase Ti_3_C_2_T_x_. **c** SEM image of Ti_3_C_2_T_x_. **d** Preparation of MXene Ti_3_C_2_T_x_. **e** XRD patterns of Ti_3_AlC_2_ and Ti_3_C_2_T_x_.


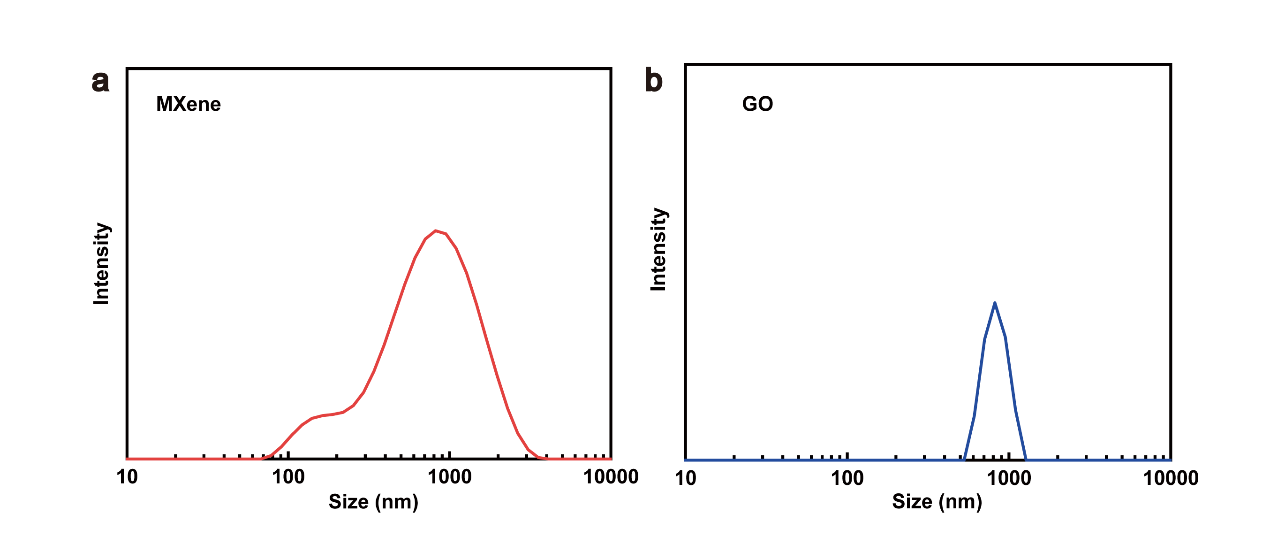


**Fig. S2** DLS size distribution diagrams of **a** MXene dispersion, and **b** GO dispersion.


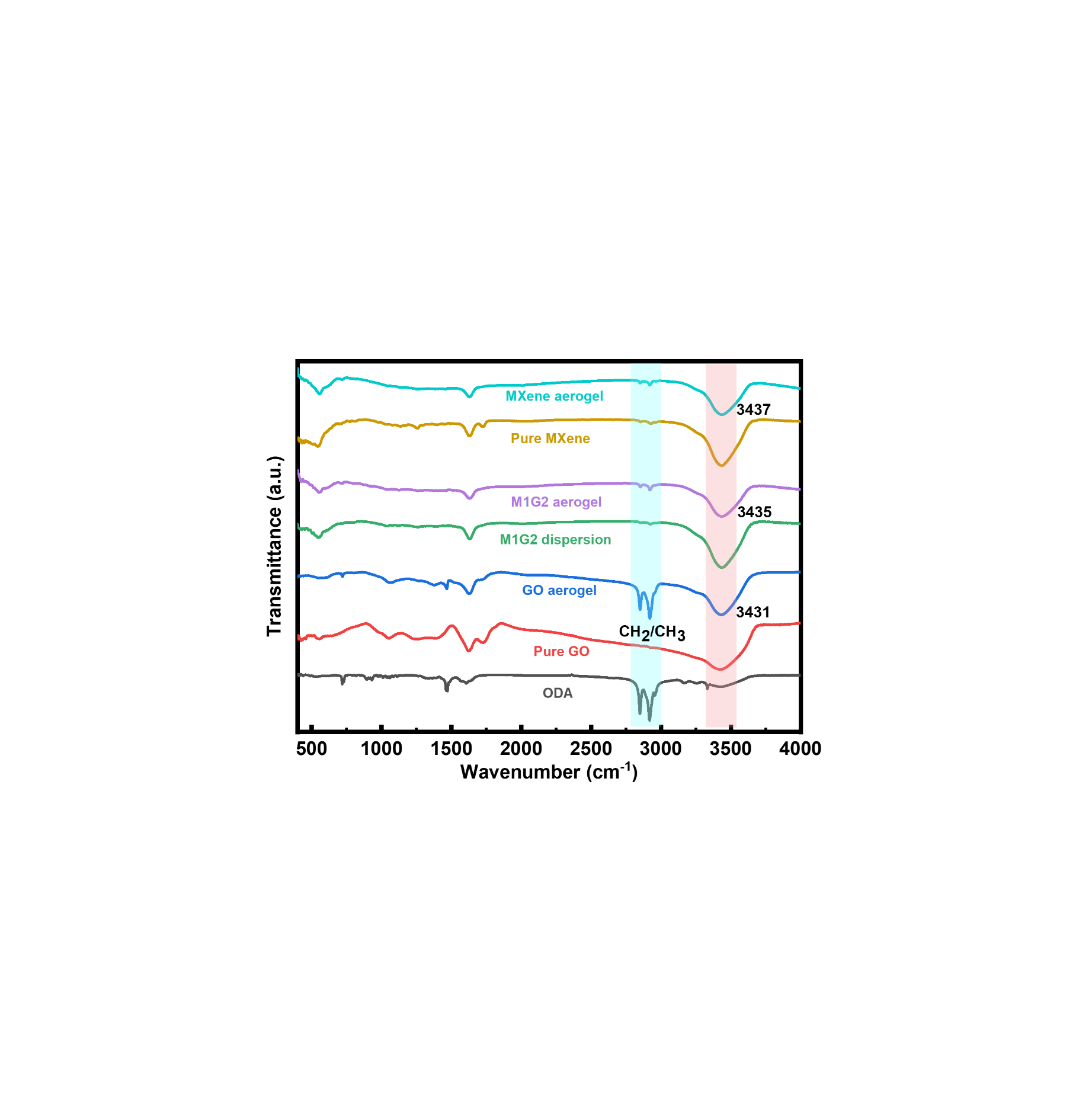


**Fig. S3** FTIR spectra of ODA, GO, MXene, and aerogels.


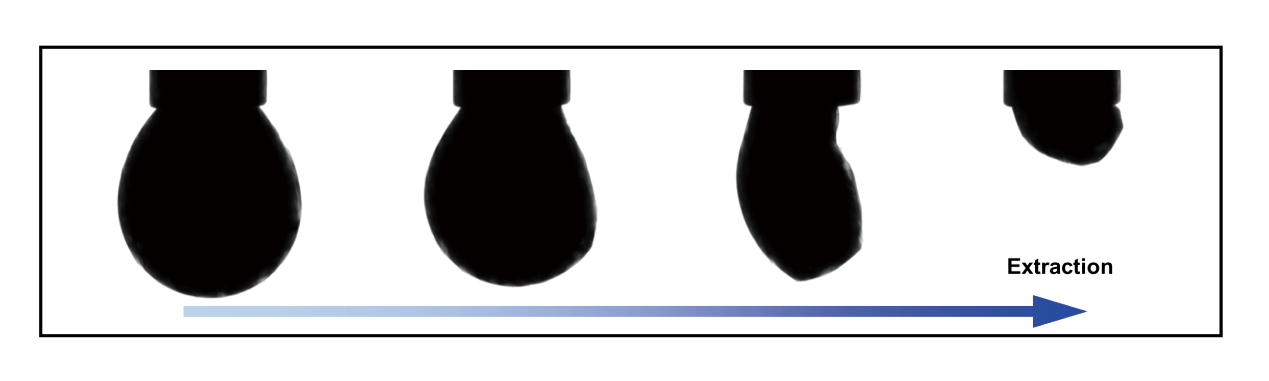


**Fig. S4** Digital photos of contracting a pendent drop of an aqueous MXene suspension (10 mg mL^-1^) in the ODA/toluene solution (10 mg mL^-1^).


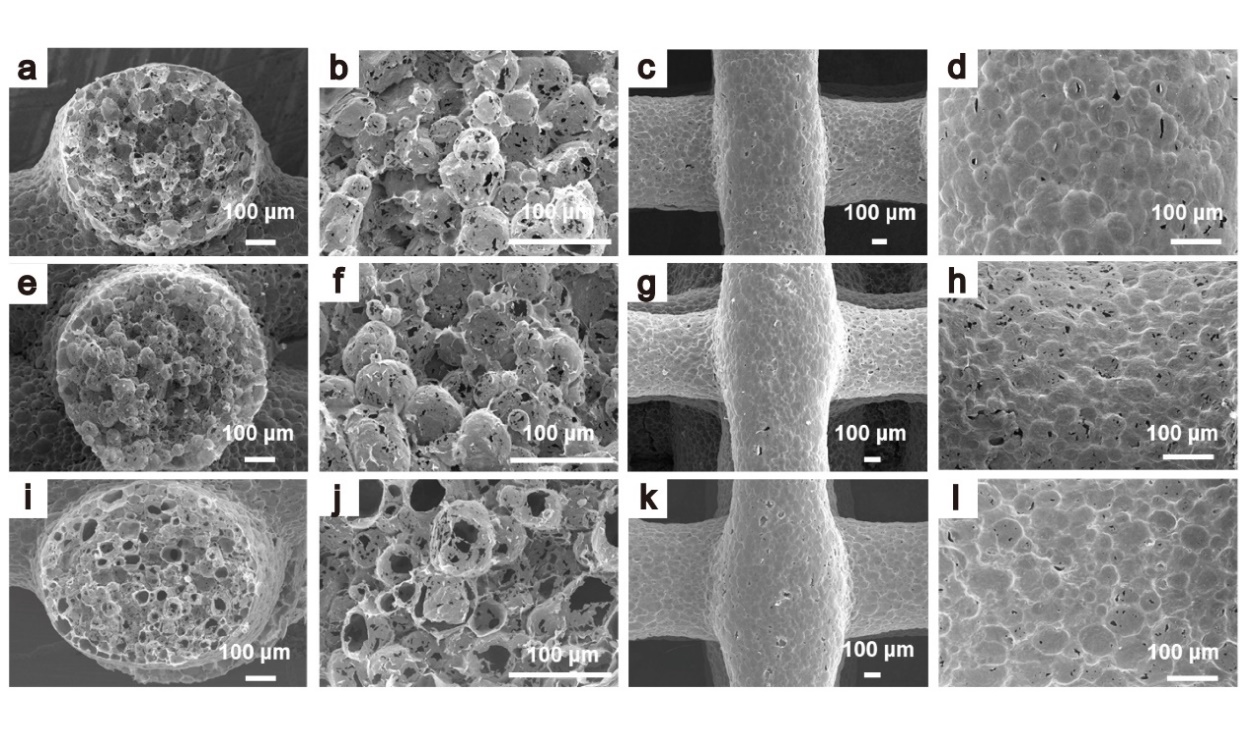


**Fig. S5** SEM images of **a-d** M2G1 aerogel, **e-h** M1G1 aerogel, and **i-l** M1G2 aerogel.


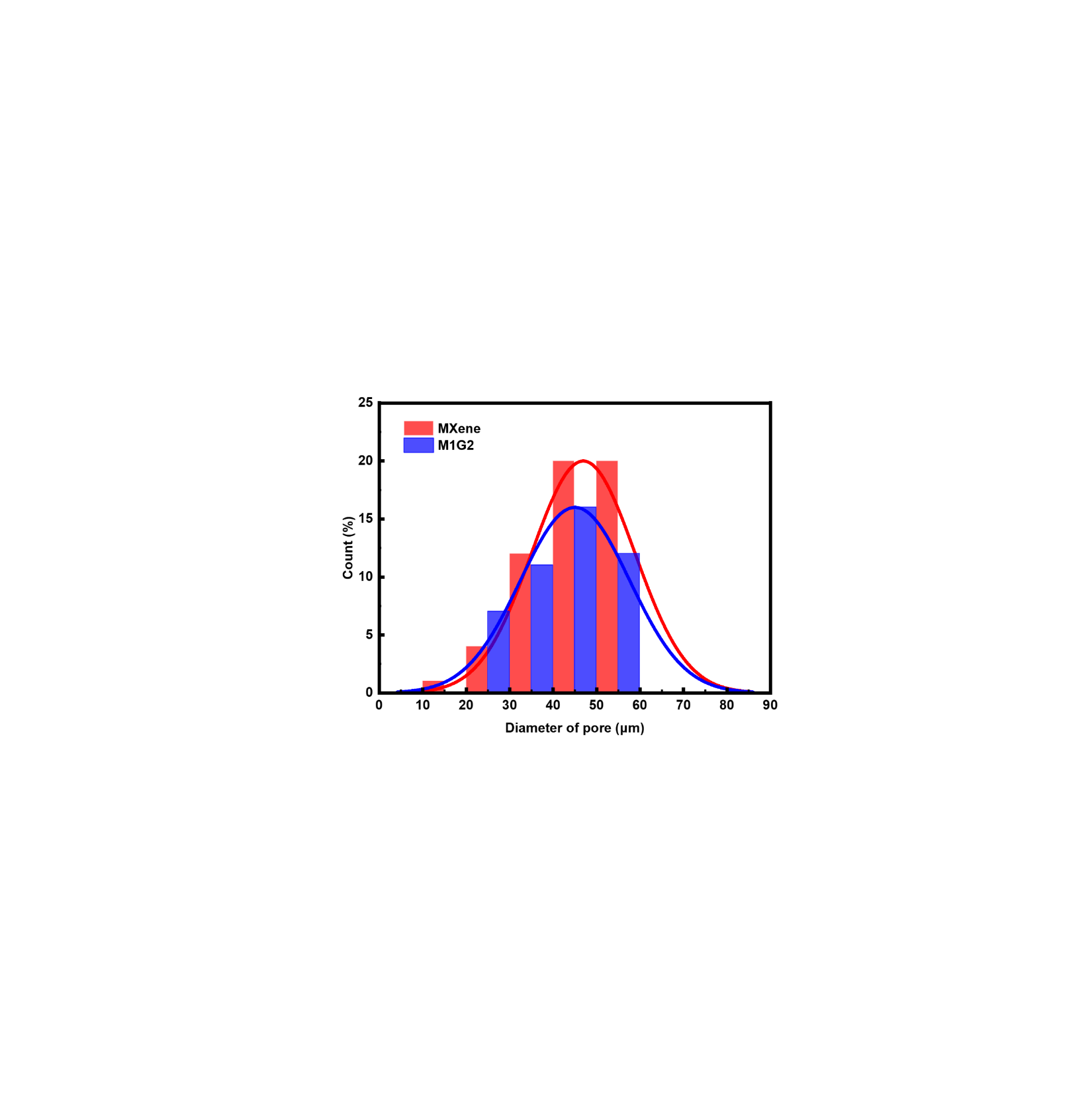


**Fig. S6** Pore sizes of MXene aerogel, and M1G2 aerogel.


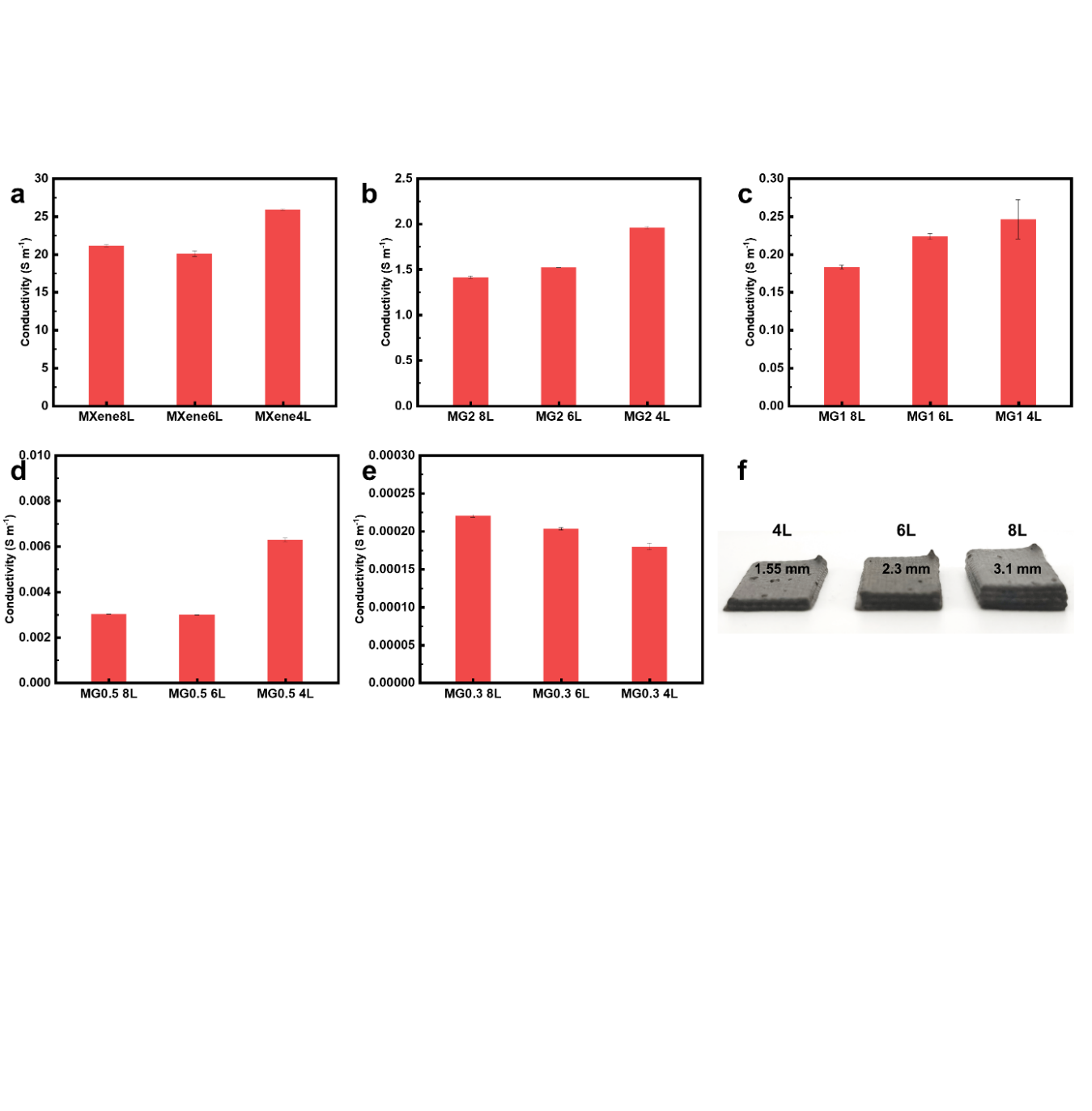


**Fig. S7** Conductivities of **a** MXene aerogel, **b** MG2 aerogel, **c** MG1 aerogel, **d** MG0.5 aerogel, and **e** MG0.3 aerogel. **f** Thicknesses of aerogels with different printed layers.


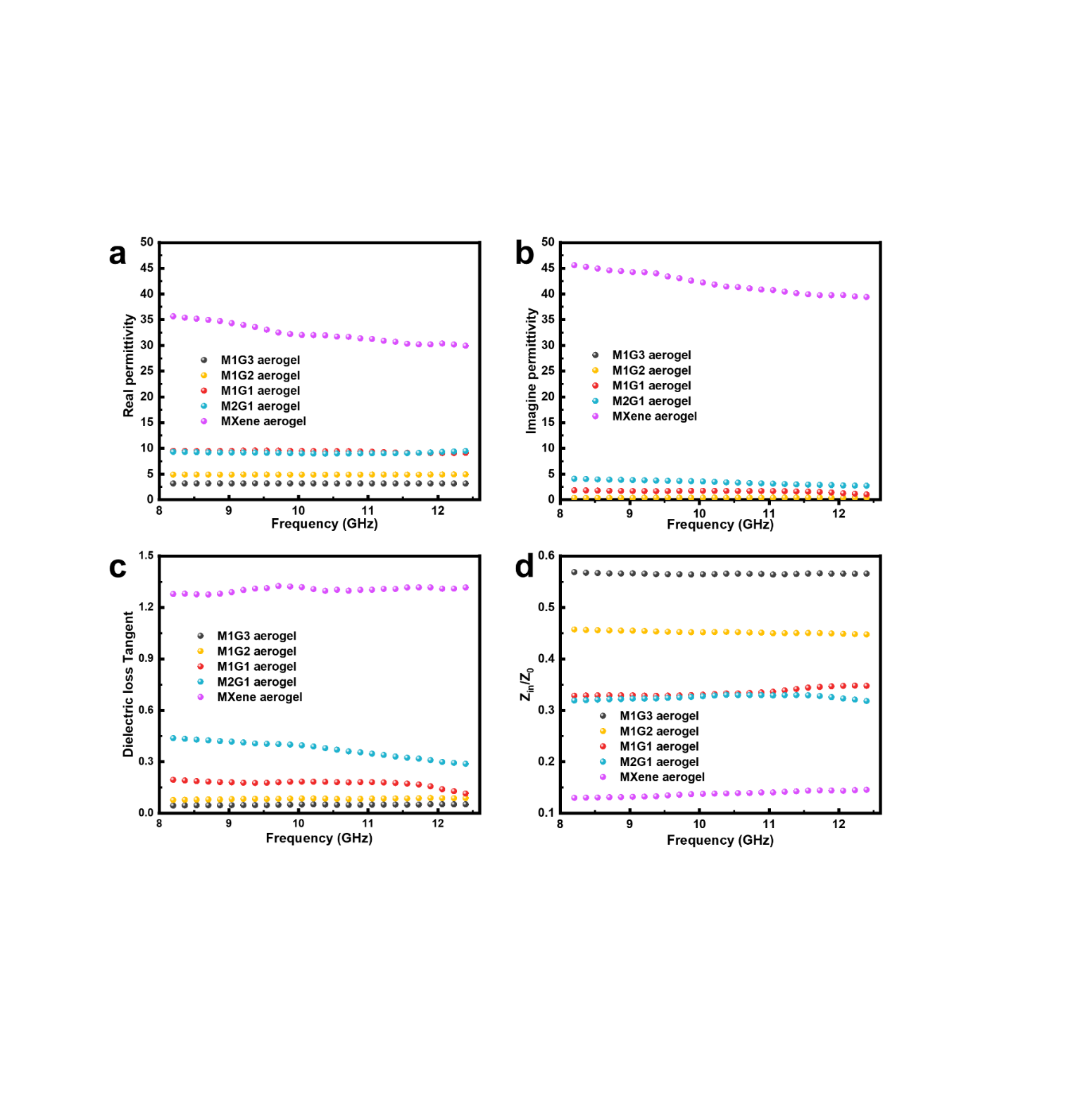


**Fig. S8** Plots of **a** real permittivity, **b** imaginary permittivity, **c** tangent loss, and **d** impedance matching of MXene, M2G1, M1G1, M1G2, and M1G3 aerogels.


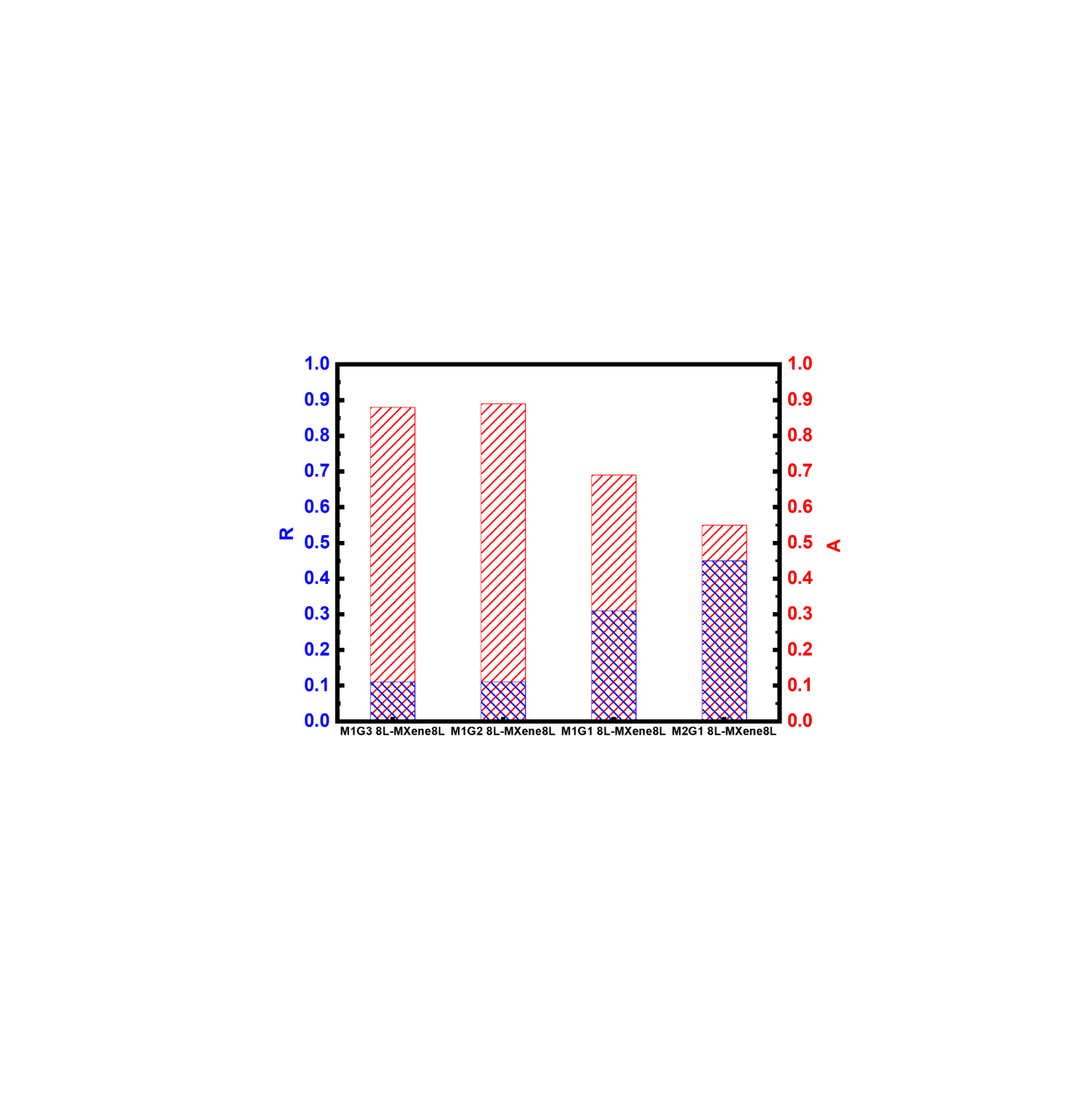


**Fig. S9** Plots of average absorption coefficient (A) and reflection coefficient (R) of MG8L-MXene8L aerogels with different GO contents in the X-band.


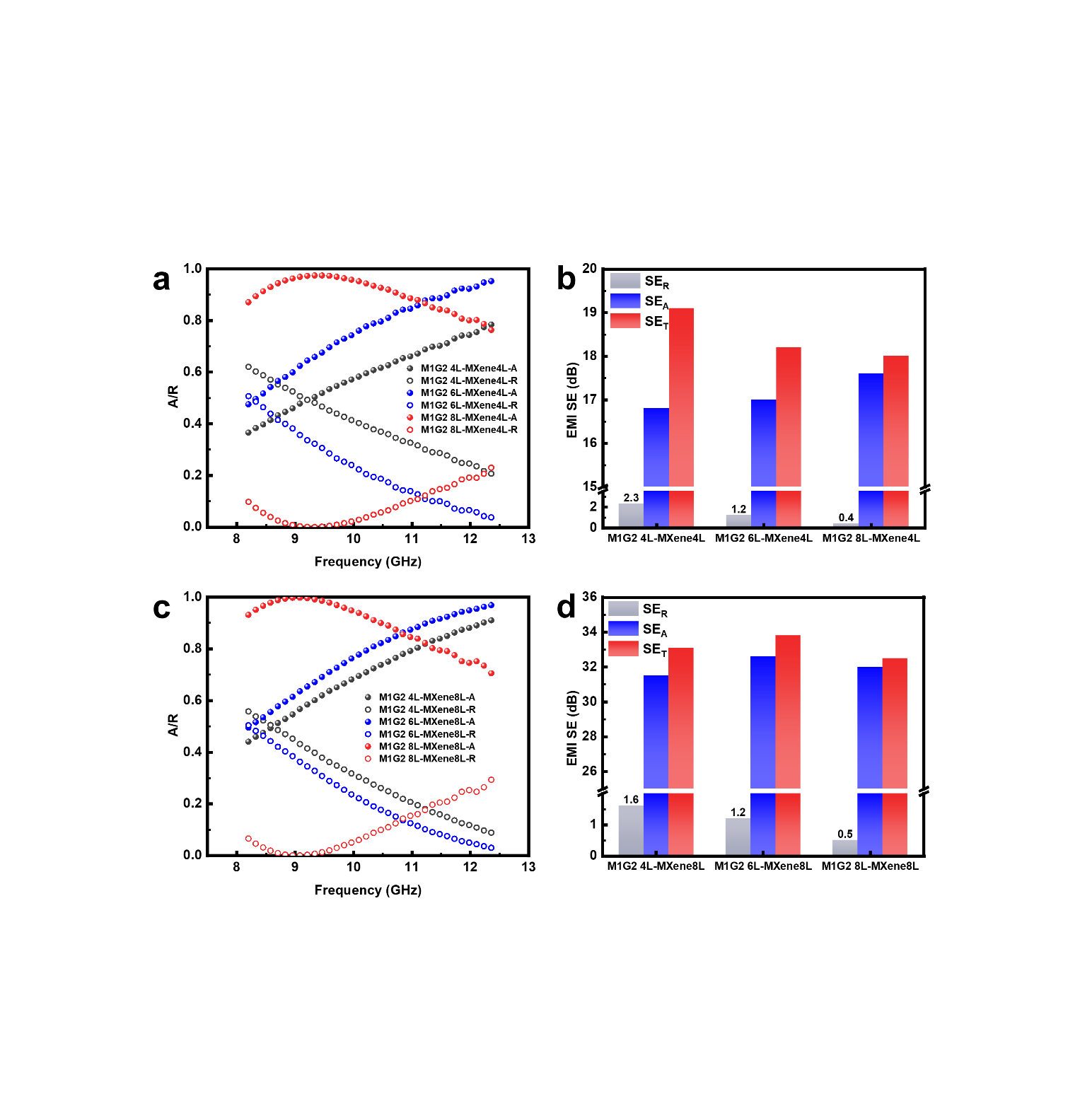


**Fig. S10** Plots of **a** A and R, and **b** EMI SE of M1G2-MXene4L aerogels with different thicknesses of MG layers. Plots of **c** A and R, and **d** EMI SE of M1G2-MXene8L aerogels with different thicknesses of MG layers.


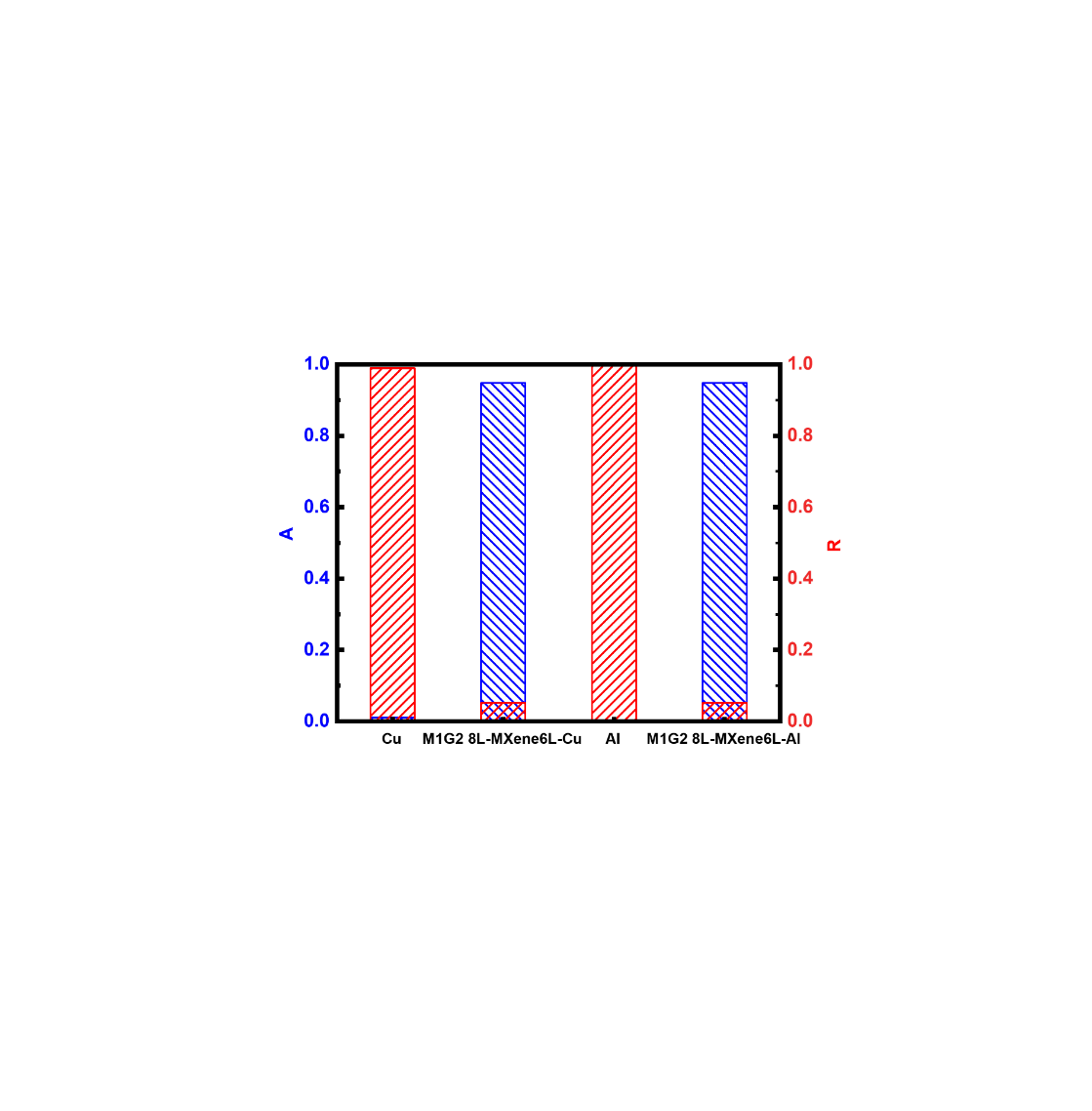


**Fig. S11** Average A and R values of copper foil and aluminum foil in the X-band, and average A and R values of M1G2 8L-MXene6L-Cu and M1G2 8L-MXene6L-Al in the X-band.


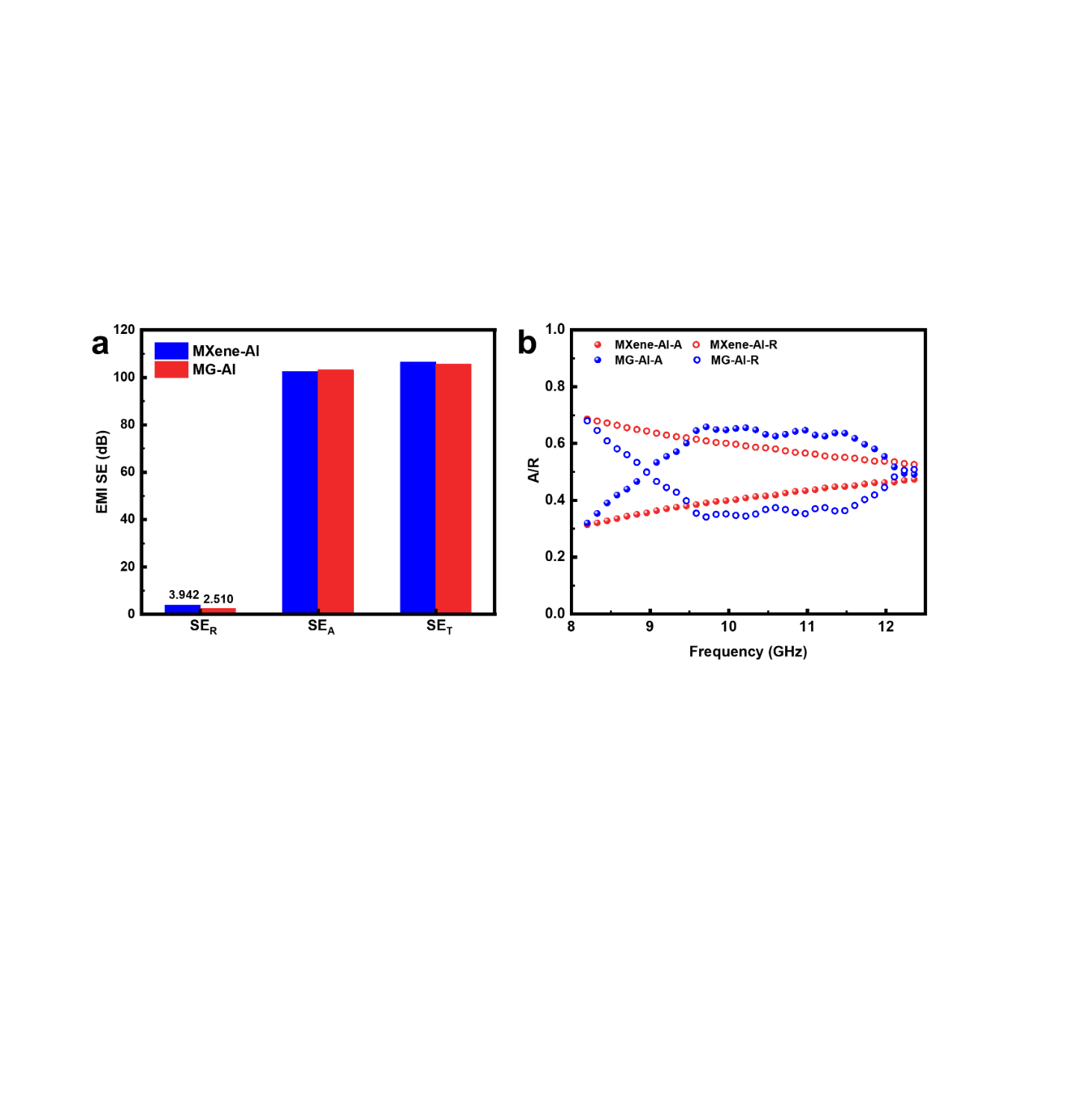


**Fig. S12** **a** Plots of SE_R_, SE_A,_ and SE_T_ of MXene-Al and MG-Al in the X-band. **b** Plots of A and R of MXene-Al and MG-Al in the X-band.


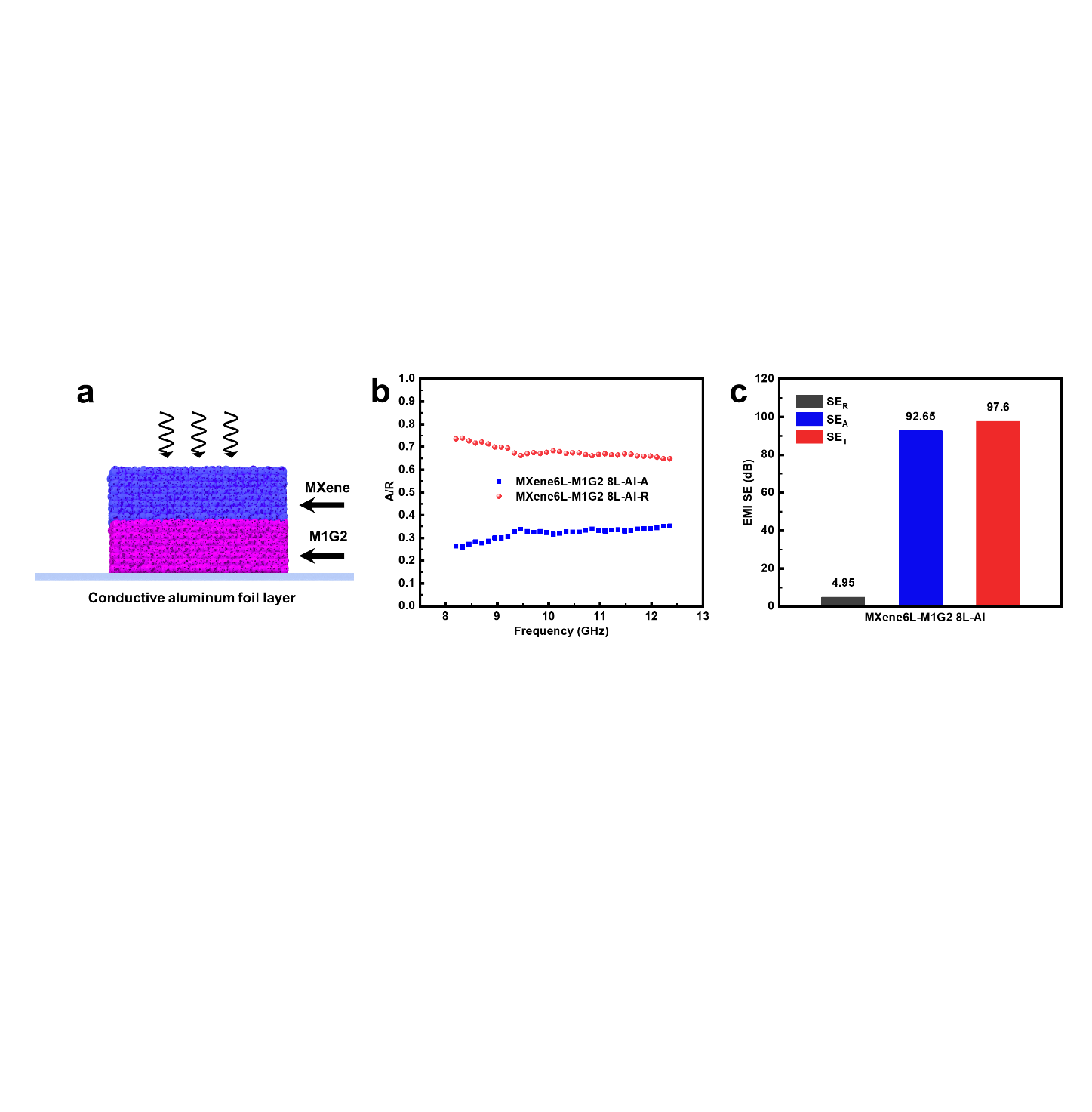


**Fig. S13** **a** Schematic diagram of electromagnetic wave incident direction of MXene6L-M1G2 8L-Al aerogel. Plots of **b** A and R, and **c** average EMI SE of MXene6L-M1G2 8L-Al in the X-band.


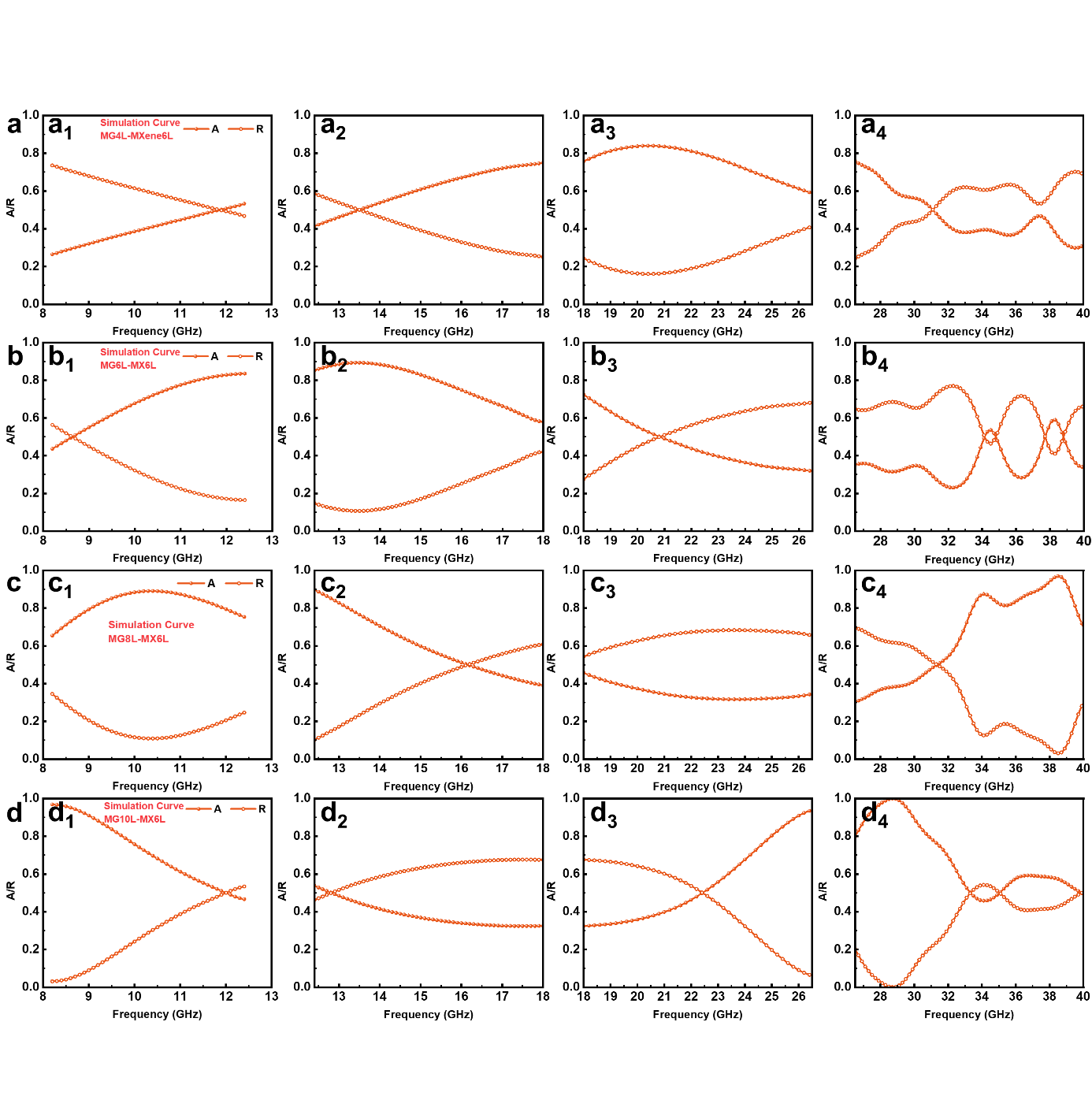


**Fig. S14** Simulation curves of A and R of M1G2-MXene with different thickness of M1G2 layer in 8.2-40 GHz band. Simulation curves of A and R of **a** M1G2 4L-MXene6L, **b** M1G2 6L-MXene6L, **c** M1G2 8L-MXene6L, and **d** M1G2 10L-MXene6L.


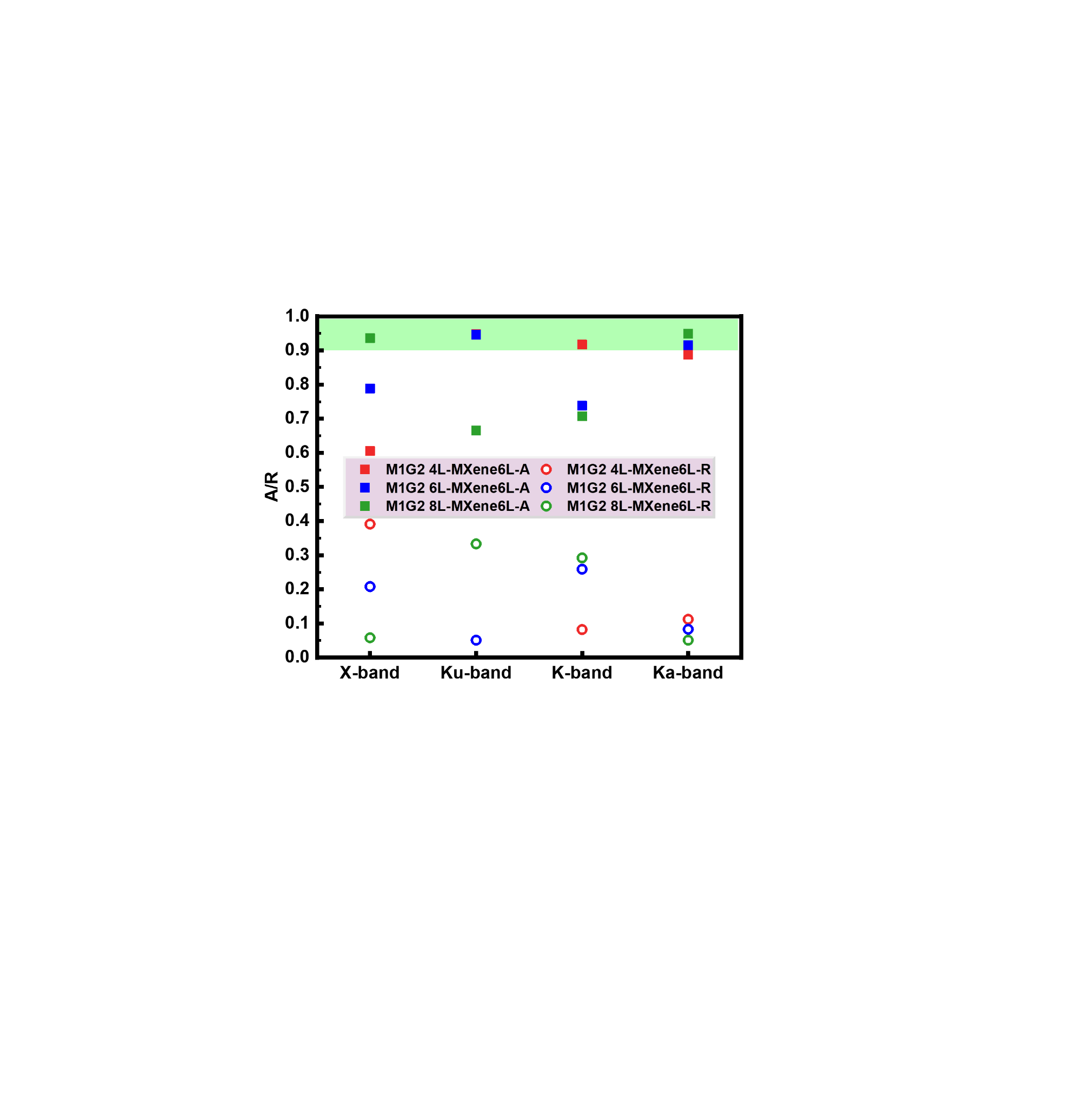


**Fig. S15** A and R values of MG-MXene aerogels in different bands.


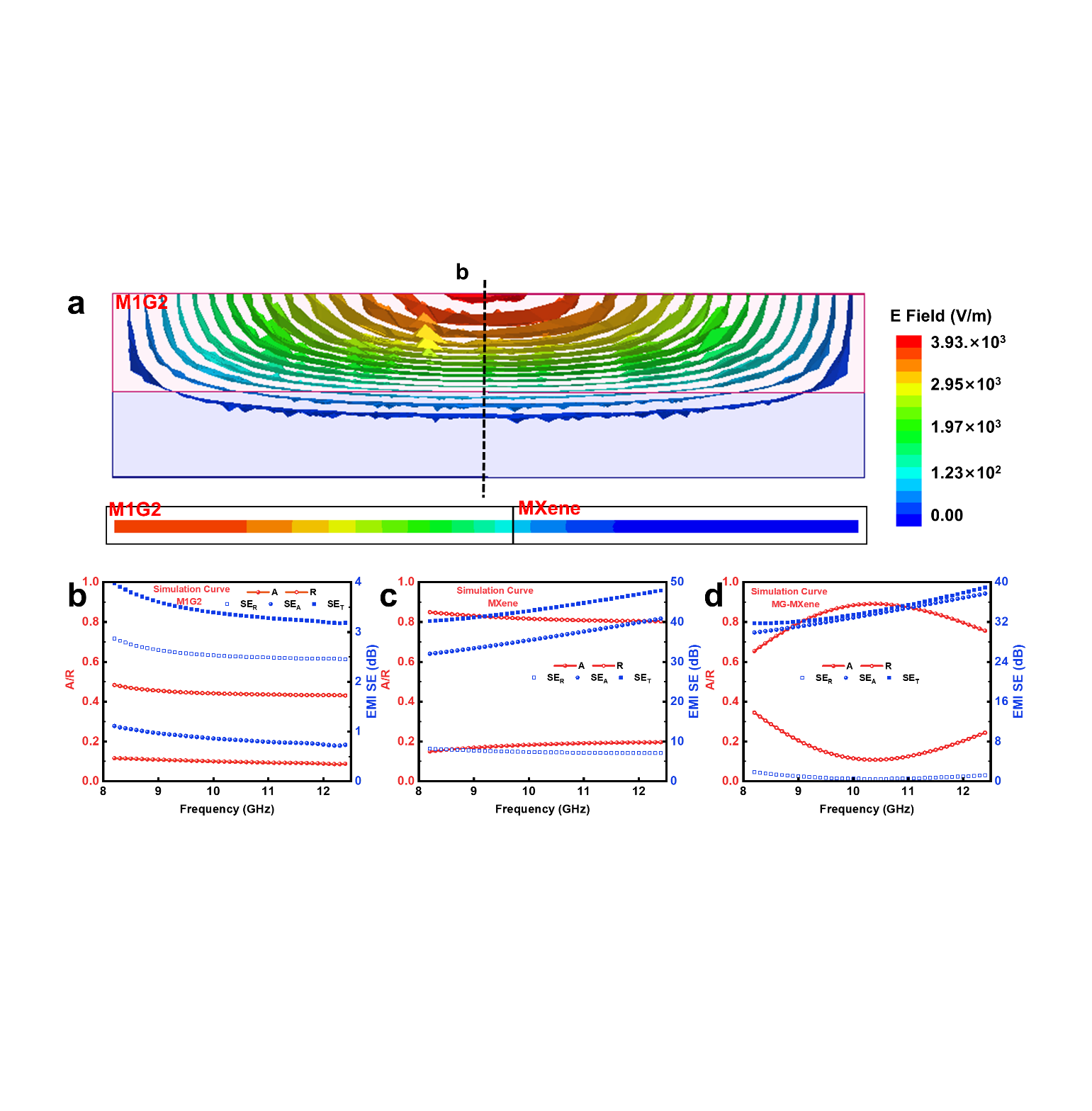


**Fig. S16** **a** Simulated electric field distribution and **b** cross-section at the dotted line for M1G2-MXene dense structure. Simulation curves of R and A of **c** M1G2 dense layer, **d** MXene dense layer, and e M1G2-MXene dense structure.


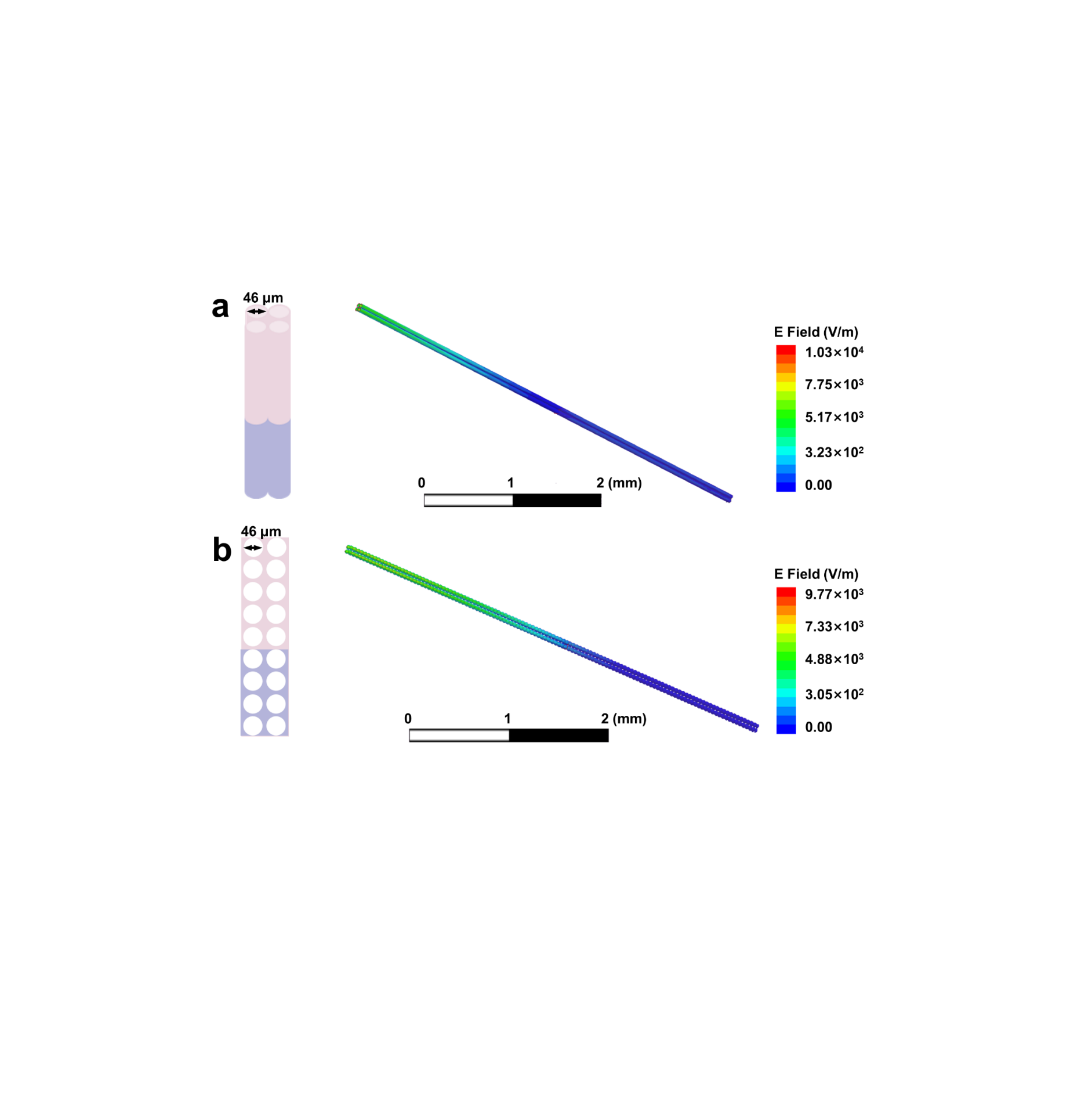


**Fig. S17** Electric field simulation distributions of **a** straight pore structure and **b** spherical pore structure.


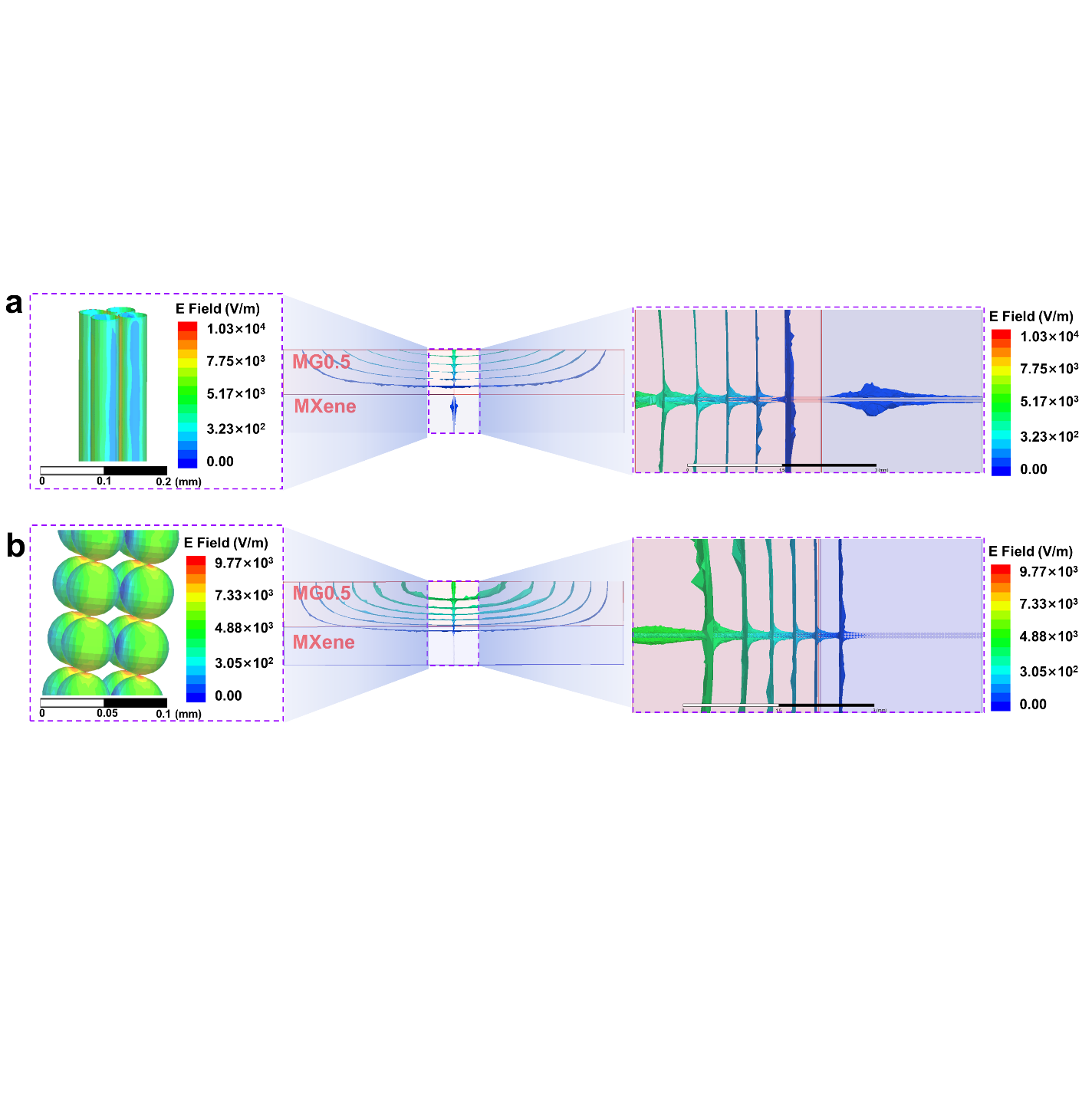


**Fig. S18** Simulated electric field distributions of **a** straight pore structure and **b** spherical pore structure.


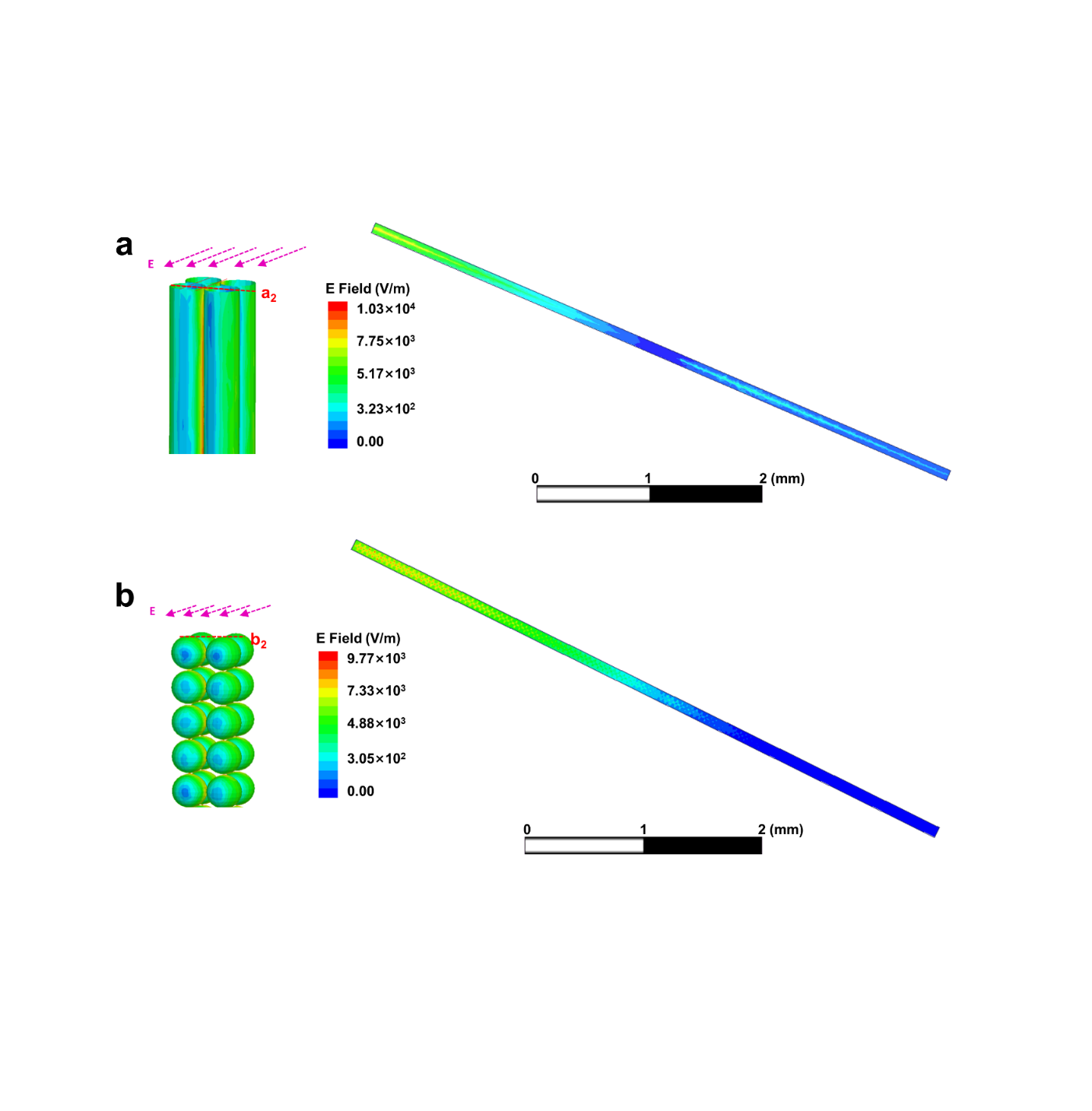


**Fig. S19** **a** Simulated electric field distribution of **a_2_** the vertical dashed plane of MG-MXene straight pore structure. **b** Simulated electric field distribution of **b_2_** the vertical dashed plane of MG-MXene spherical pore structure.


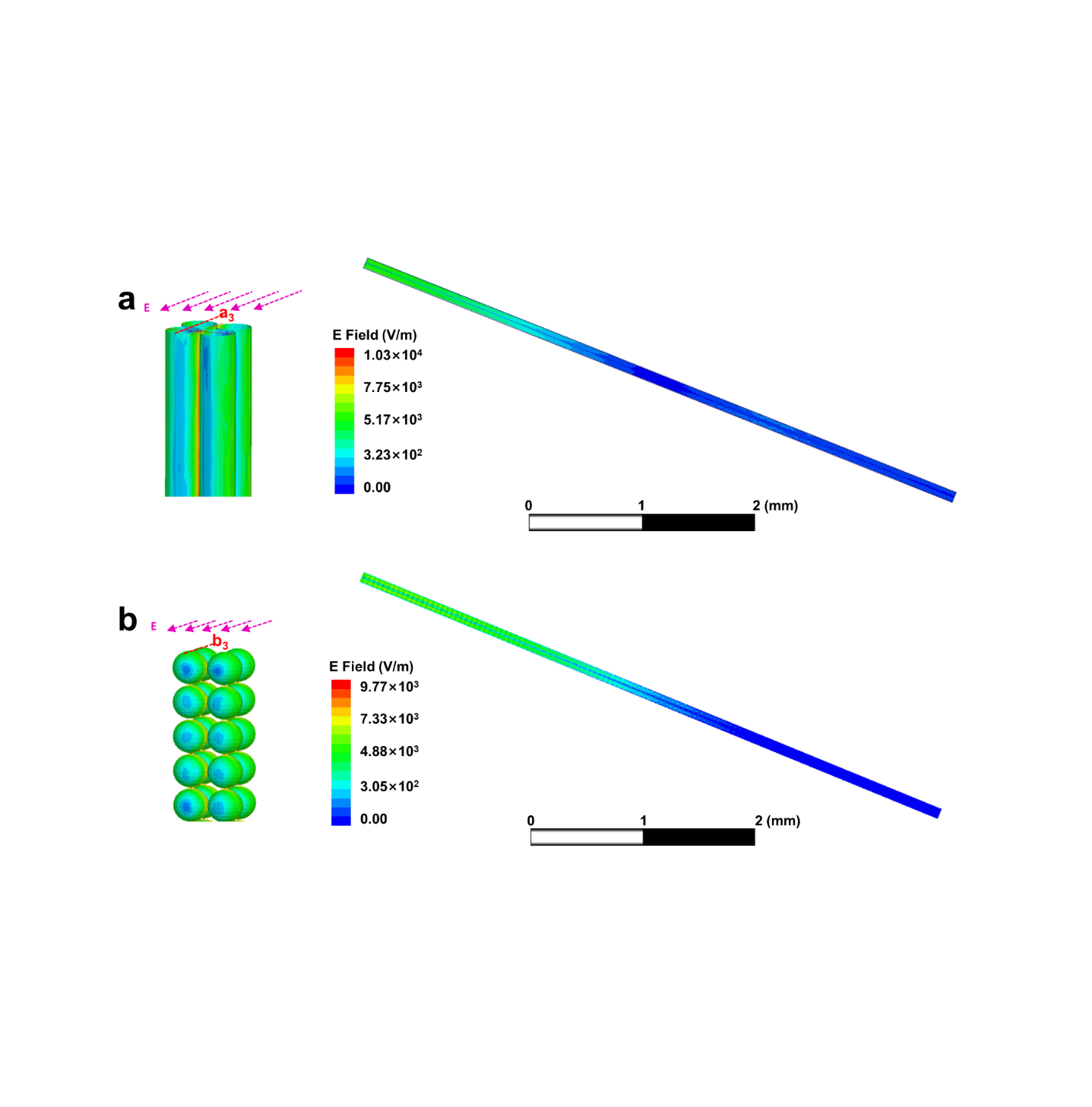


**Fig. S20** **a** Simulated electric field distribution of **a_3_** the vertical dashed plane of MG-MXene straight pore structure. **b** Simulated electric field distribution of **b_3_** the vertical dashed plane of MG-MXene spherical pore structure.


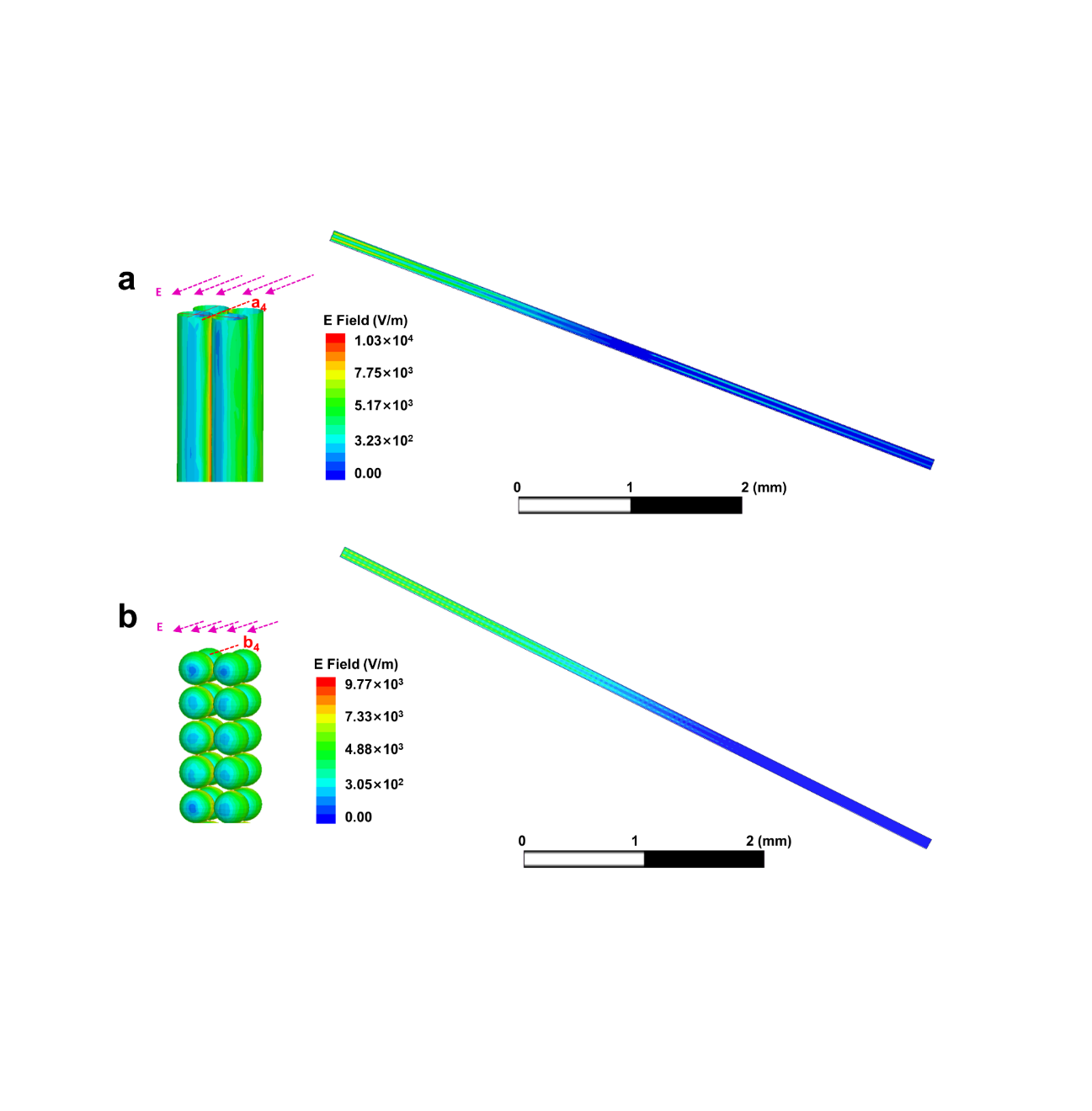


**Fig. S21** **a** Simulated electric field distribution for **a_4_** the vertical dashed plane of MG-MXene straight pore structure. **b** Simulated electric field distribution for **b_4_** the vertical dashed plane of MG-MXene spherical pore structure.


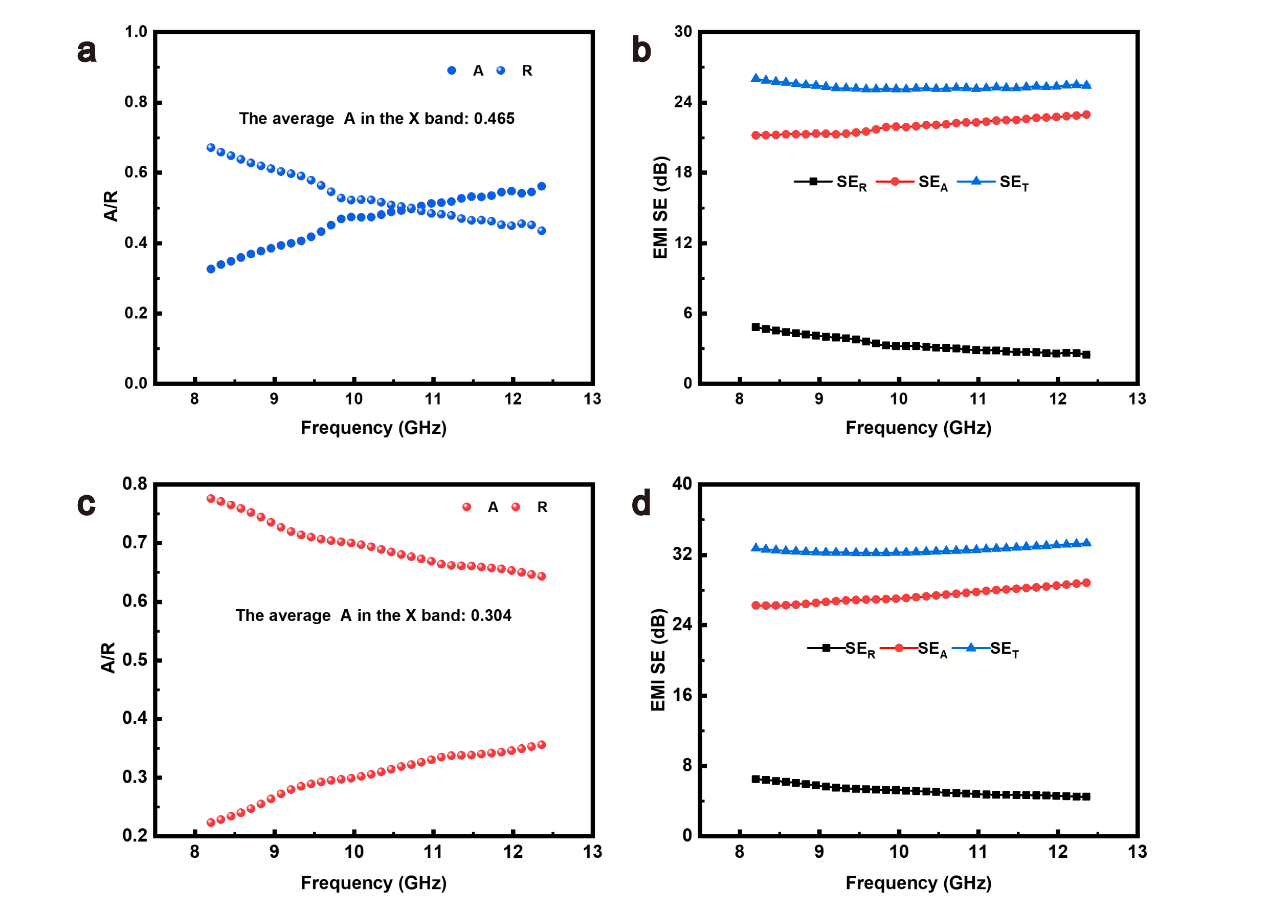


**Fig. S22** Plots of **a** A and R, and **b** EMI SE of the M1G2-MXene aerogel prepared by random freezing. Plots of **c** A and R, and **d** Plots of EMI SE of the M1G2-MXene aerogel prepared by directional-freezing.


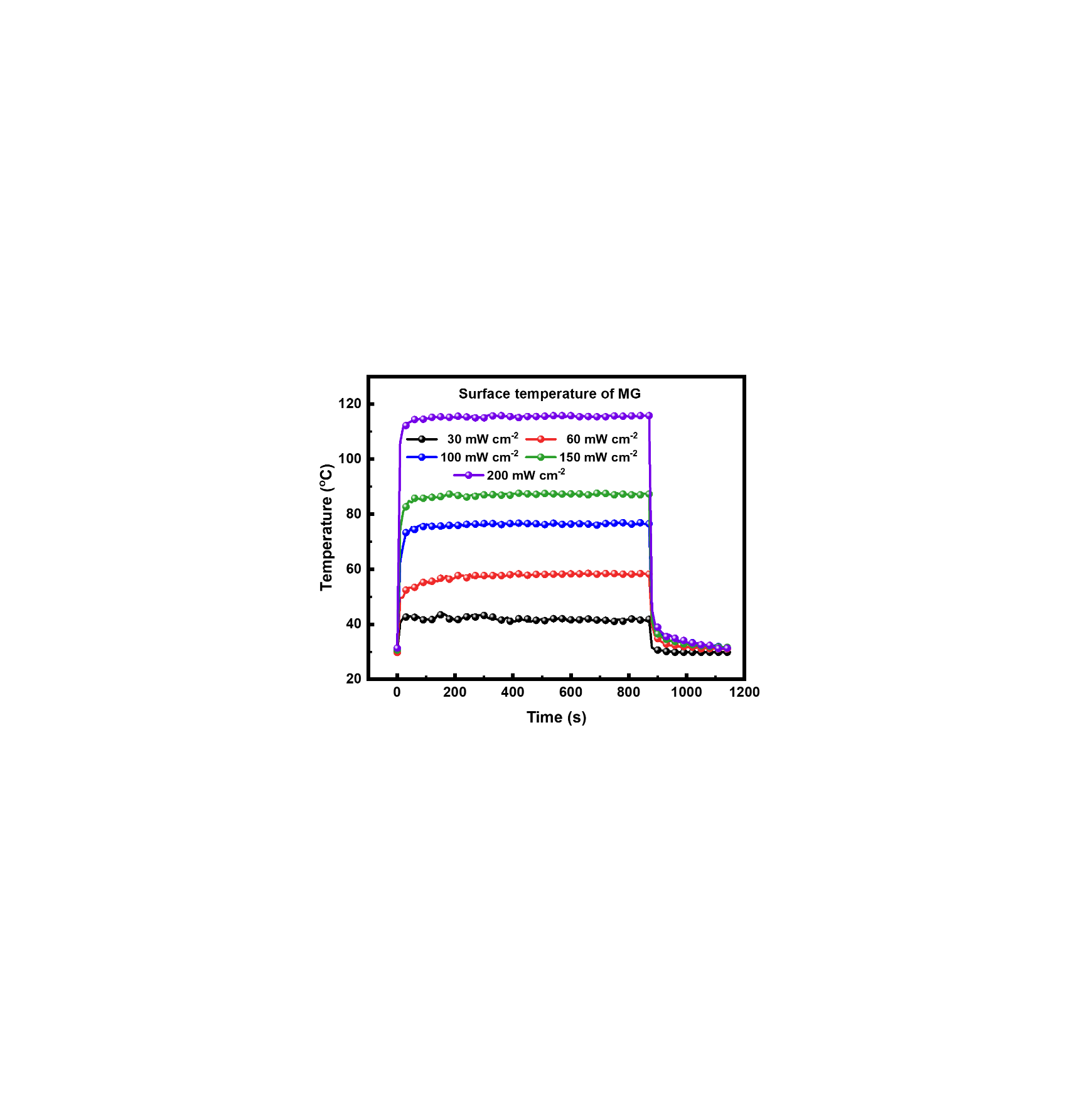


**Fig. S23** Solar-thermal energy conversion curves of the MG-MXene aerogel at different solar light power densities.


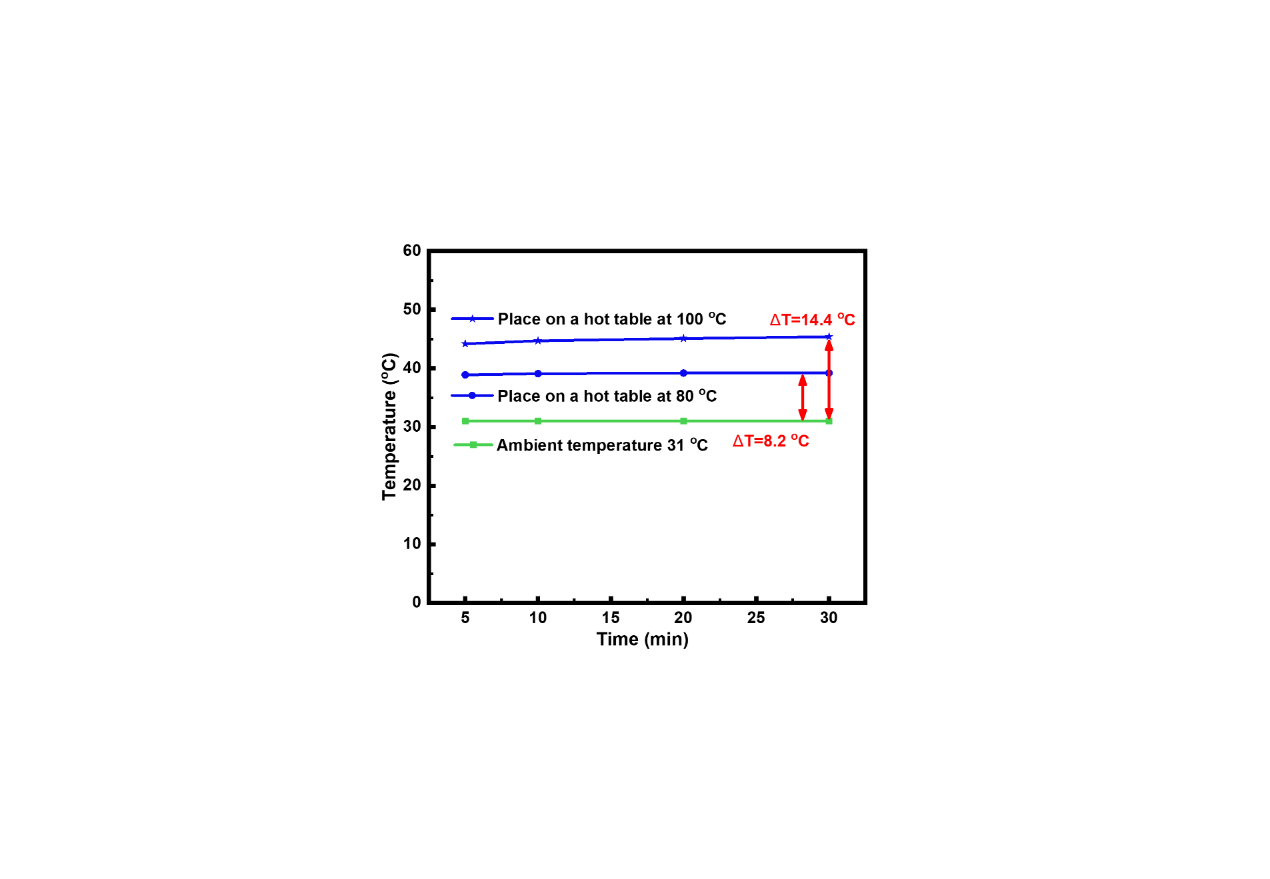


**Fig. S24** Top surface temperatures of the MG-MXene aerogel placing on a heating platform of 80 and 100 °C.

**Table S1** Compositions of 3D printing aerogels.

| Samples | Components, Concentration (mg mL^-1^), and Ratios |
| --- | --- |
| MXene | MXene (15 mg mL^-1^), 1 |
| M2G1 | MXene (15 mg mL^-1^)/GO (7.5 mg mL^-1^), 2/1 |
| M1G1 | MXene (15 mg mL^-1^)/GO (7.5 mg mL^-1^), 1/1 |
| M1G2 | MXene (15 mg mL^-1^)/GO (7.5 mg mL^-1^), 1/2 |
| M1G3 | MXene (15 mg mL^-1^)/GO (7.5 mg mL^-1^), 1/3 |

**Table S2** Comparison of EMI shielding performances of 3D printing aerogels

| Samples | Density  (mg cm^-3^) | Thickness  (mm) | EMI SE  (dB) | A | Band | Refs. |
| --- | --- | --- | --- | --- | --- | --- |
| M1G2 8L-MXene6L | 11.83 | 5.6 | 23.2 | 0.95 | X-band | This work |
|  |  |  | 43.7 | 0.95 | Ka-band | This work |
| M1G2 6L-MXene6L | 11.60 | 4.8 | 24.9 | 0.95 | Ku-band | This work |
| M1G2 4L-MXene6L | 11.76 | 4.0 | 32.1 | 0.92 | K-band | This work |
| M1G2 3-MXene2 | 10.00 | 5.0 | 18.4 | 0.95 | X-band | This work |
| M1G2 8L-MXene6L-Cu | / | 5.6 | 100.7 | 0.95 | X-band | This work |
| M1G2 8L-MXene6L-Al | / | 5.6 | 106.1 | 0.95 | X-band | This work |
| MXene/CNT/PI aerogel | 152.00 | 5 | 68.2 | 0.77 | X-band | [S1] |
| PLA/Ag | 262.00 | 1.5 | 110 | 0.79 | X-band | [S2] |
| rGO@Fe_3_O_4_/T-ZnO /Ag/WPU | / | 0.5 | 87.2 | 0.61 | X-band | [S3] |
| PI/graphene aerogel | 83.96 | 2.5 | 28.8 | 0.63 | X-band | [S4] |
| NG@Fe_3_O_4_  /PEDOT:PSS | ~100.00 | 5.0 | 54.0 | 0.59 | 12.4 GHz | [S5] |
| M6-FC2 | 10.00 | 2 | 32.5 | 0.69 | X-band | [S6] |
| CNT/RGO foam | 57.20 | 2 | 31.2 | 0.26 | X-band | [S7] |
| AgNW/PDMS aerogel | 6.20 | 2 | 70.5 | 0.02 | X-band | [S8] |
| G@Fe_3_O_4_/PEI aerogel | 433.73 | 2.5 | 18.2 | 0.89 | X-band | [S9] |

**References**

[S1] T. Xue, Y. Yang, D. Yu, Q. Wali, Z. Wang et al., 3D printed integrated gradient-conductive MXene/CNT/polyimide aerogel frames for electromagnetic interference shielding with ultra-low reflection. Nano-Micro Lett. **15**, 45 (2023). <https://doi.org/10.1007/s40820-023-01017-5>

[S2] Y.-N. Gao, Y. Wang, T.-N. Yue, B. Zhao, R. Che et al., Superstructure silver micro-tube composites for ultrahigh electromagnetic wave shielding. Chem. Eng. J. **430**, 132949 (2022). <https://doi.org/10.1016/j.cej.2021.132949>

[S3] Y. Xu, Y. Yang, D. X. Yan, H. Duan, G. Zhao, Y. Liu, Gradient structure design of flexible waterborne polyurethane conductive films for ultraefficient electromagnetic shielding with low reflection characteristic. ACS Appl. Mater. Interfaces **10**, 19143-19152 (2018). <https://doi.org/10.1021/acsami.8b05129>

[S4] Z. Yu, T. Dai, S. Yuan, H. Zou, P. Liu, Electromagnetic interference shielding performance of anisotropic polyimide/graphene composite aerogels. ACS Appl. Mater. Interfaces **12**, 30990-31001 (2020). <https://doi.org/10.1021/acsami.0c07122>

[S5] X.-X. Wang, J.-C. Shu, W.-Q. Cao, M. Zhang, J. Yuan et al., Eco-mimetic nanoarchitecture for green emi shielding. Chem. Eng. J. **369**, 1068-1077 (2019). <https://doi.org/10.1016/j.cej.2019.03.164>

[S6] Z. Ma, Z. Deng, X. Zhou, L. Li, C. Jiao et al., Multifunctional and magnetic mxene composite aerogels for electromagnetic interference shielding with low reflectivity. Carbon **213**, 118260 (2023). <https://doi.org/10.1016/j.carbon.2023.118260>

[S7] L. Kong, X. Yin, H. Xu, X. Yuan, T. Wang et al., Powerful absorbing and lightweight electromagnetic shielding CNTs/RGO composite. Carbon **145**, 61-66 (2019). <https://doi.org/10.1016/j.carbon.2019.01.009>

[S8] Z. Zeng, T. Wu, D. Han, Q. Ren, G. Siqueira et al., Ultralight, flexible, and biomimetic nanocellulose/silver nanowire aerogels for electromagnetic interference shielding. ACS Nano **14**, 2927-2938 (2020). <https://doi.org/10.1021/acsnano.9b07452>

[S9] B. Shen, W. Zhai, M. Tao, J. Ling, W. Zheng, Lightweight, multifunctional polyetherimide/graphene@Fe_3_O_4_ composite foams for shielding of electromagnetic pollution. ACS Appl. Mater. Interfaces **5**, 11383-11391 (2013). <https://doi.org/10.1021/am4036527>
